# Analytical methods for identifying sequences of utilization in health data: a scoping review

**DOI:** 10.1186/s12874-023-02019-y

**Published:** 2023-09-27

**Authors:** Amelie Flothow, Anna Novelli, Leonie Sundmacher

**Affiliations:** Chair of Health Economics, Technical University of Munich, Georg-Brauchle-Ring, Munich, Bavaria 80992 Germany

**Keywords:** Health data, Claims data, Care pathway, Patient pathway, Pattern mining, Data mining method, Sequences, Scoping review

## Abstract

**Background:**

Healthcare, as with other sectors, has undergone progressive digitalization, generating an ever-increasing wealth of data that enables research and the analysis of patient movement. This can help to evaluate treatment processes and outcomes, and in turn improve the quality of care. This scoping review provides an overview of the algorithms and methods that have been used to identify care pathways from healthcare utilization data.

**Method:**

This review was conducted according to the methodology of the Joanna Briggs Institute and the Preferred Reporting Items for Systematic Reviews Extension for Scoping Reviews (PRISMA-ScR) Checklist. The PubMed, Web of Science, Scopus, and EconLit databases were searched and studies published in English between 2000 and 2021 considered. The search strategy used keywords divided into three categories: the method of data analysis, the requirement profile for the data, and the intended presentation of results. Criteria for inclusion were that health data were analyzed, the methodology used was described and that the chronology of care events was considered. In a two-stage review process, records were reviewed by two researchers independently for inclusion. Results were synthesized narratively.

**Results:**

The literature search yielded 2,865 entries; 51 studies met the inclusion criteria. Health data from different countries ($n=12$) and of different types of disease ($n=26$) were analyzed with respect to different care events. Applied methods can be divided into those identifying subsequences of care and those describing full care trajectories. Variants of pattern mining or Markov models were mostly used to extract subsequences, with clustering often applied to find care trajectories. Statistical algorithms such as rule mining, probability-based machine learning algorithms or a combination of methods were also applied. Clustering methods were sometimes used for data preparation or result compression. Further characteristics of the included studies are presented.

**Conclusion:**

Various data mining methods are already being applied to gain insight from health data. The great heterogeneity of the methods used shows the need for a scoping review. We performed a narrative review and found that clustering methods currently dominate the literature for identifying complete care trajectories, while variants of pattern mining dominate for identifying subsequences of limited length.

**Supplementary Information:**

The online version contains supplementary material available at 10.1186/s12874-023-02019-y.

## Background

Throughout the world, increasing quantities of data are being produced, collected, stored and processed on a continuous basis. This affects the most diverse areas of life. Following the approach of the International Data Corporation (IDC) [1] the healthcare datasphere was found to be the sixth largest enterprise datasphere in the world in 2018. The storage volume amounted to 1, 218 exabyte, which corresponds to $1,218~\times ~10^{9}$ gigabyte. It is expected that the growth rate of the healthcare datasphere will reach 36 % by 2025. This is higher than the growth of the global data sphere in all other industries.

The health care sector is characterized by routine data collection, leading to its accumulation in large databases. This includes, for example, data collected by health insurance companies for billing purposes [2], treatment data in hospitals, and databases of health data aggregated at a national or subnational level [3]. Furthermore, the increased use of technology and digital health applications open up a new pool of health care data which may accelerate the growth in data volume. By applying advanced algorithms to this large corpus of data, the structured study and analysis of health data has great potential to provide valuable insight into care processes. In particular, the use of extensive real-life care data is a promising approach for the identification of care pathways. A care pathway, alternatively termed a sequence or trajectory, is constructed as a succession of (different) care events that take place within a fixed time horizon. If only part of the fixed time horizon is considered, we call it a subsequence or pattern, knowing that all sequences can also be interpretated as subsequences of a longer time horizon. Sequences of care can include events such as physician visits, the recording of a diagnosis, procedures such as blood tests or non-invasive diagnostics, outpatient or inpatient surgeries, as well as medication prescription or emergency treatment at a hospital. Most care events correspond to health care utilized by the patient. Health care data collected in the ambulatory sector record the utilization behaviour of the patient and the treatment provided, which in turn is influenced by the recommendations of the physicians consulted. Most importantly, this enables the identification and analysis of healthcare pathways. Exploratory analysis of such pathways, sequences, or patterns in comprehensive data can contribute to insight in multiple ways. It provides healthcare providers, researchers, and policymakers with a holistic view of the care situation of the patient population under study [4–6]. Depending on the time horizon, it allows, for example, to investigate the continuity of long-term therapies and healthcare use of chronically ill patients [4, 6, 7], or the inpatient pathways of hospitalization [8, 9]. Comparison of identified empirical, real-world sequences with ideal, normative pathways or treatment recommendations from evidence-based treatment guidelines can help identify gaps in care and deviations from desired care pathways. Correlations with patient characteristics and supply-side factors can provide an even better picture of healthcare delivery in the patient population studied and identify starting points for further research [4, 5, 9]. The correlation of identified pathways with health outcomes is relevant for formulating care recommendations and improving or informing the design of normative pathways [9, 10]. When deviations from guidelines are identified, they can be targeted for interventions. In addition, healthcare pathway analysis can be used to estimate future costs and anticipate needed resources [6].

Advanced algorithms make it possible to analyze the huge amount of existing health care data and to obtain real-world care pathways. The field of data analysis is very broad and is undergoing rapid growth. A range of algorithms, including machine learning techniques, are already being used to analyze health care data. This scoping review shall provide an overview of the methods previously used to identify sequences of care, focusing on utilization events in health data. Some existing literature has addressed related research questions. A literature review on clinical pathway modelling was published in 2019 [11]. It provides an overview of publications on clinical pathways in health care and was therefore limited to the clinical sector. The focus was placed on the intersection of Information Systems, Operational Research and industrial engineering. Other literature reviews focus on process mining to examine health care data. This includes a literature review with a search strategy that is strongly focused on process mining (“(“process mining” OR “workflow mining”)” [12, p. 377]) in primary care, published in 2018 [12], and a further literature review focusing again on process mining techniques (“(“process mining” OR “workflow mining”)” [13, p. 458]) to identify patterns of diseases, published in 2021 [13]. A forth publication presents an essential monitoring tool to conduct a systematic review of patients’ health care [14]. A semi-automatic text mining method was used to extract the semantic perspective of the included studies and the results were further supplemented by hand. Only articles published between year 2000 and 2005 were considered. The existing literature shows that there is great interest in the analysis of health data. However, the research presented is either limited to pre-selected types of methods or considers a very limited period of time. The methods themselves are only considered at a basic level. In contrast, this review aims to provide a broad overview of the methods used to identify care pathways using health care utilization data, focussing on those that consider the chronology of the events. The main features of the most commonly used methodological approaches are described. In particular, this scoping review conducts a systematic literature search to answer the following questions:

- What research objectives are answered in the included literature?
- What recent methods have been used to identify sequences of care in health data?
- How are the identified sequences represented?

The relations between the methodology used, the stated research objective, and the presentation of results are described. In addition, basic characteristics of the included studies are provided. This systematic review shall therefore provide a comprehensive overview of the existing literature and help researchers make informed methodological decisions for their own research. To our knowledge this is the first literature review focusing on this topic.

## Methods

A scoping review was conducted to answer the stated research questions, following the methodology of the Joanna Briggs Institute (JBI) for scoping reviews [15] and the Preferred Reporting Items for Systematic Reviews (PRISMA) Extension for Scoping Reviews (PRISMA-ScR) [16]. A study protocol was not submitted.

### Eligibility criteria

Inclusion and exclusion criteria were defined to identify relevant studies that analyze health care data and identify chronological sequences of care events (also: care pathway). In particular, to be considered as eligible a study must: 1. apply the method used on individual, patient-focused and chronological care data, 2. describe the method applied or at least name the chosen method, if the method is well-known, and 3. present the resulting care sequence considering a chronological sequence of health care utilization events. Further, the datasets had to contain individual health data on health care utilization and be collected systematically (e.g. no questionnaire or reporting). Datasets including only the disease or medical values, as well as strongly aggregated data (e.g. in a temporal way or on a patient group level) were excluded. The method used had to be explained and take into account the chronology of care events. This leads to the specification of further conditions on the applied method and on the presentation of the results. The method and the resulting care sequence must consider the chronological order of the data. There is no limitation of publication, of the number of included patients or of the origin of the data. A tabulation of the inclusion and exclusion criteria is shown in Additional file 1, Part I.

### Data sources and search strategy

To find literature to answer the stated research question, the authors developed a search string combining inclusively the synonyms of three categories. First, the data basis: relevant studies must perform their analysis on real individual patient-related data. Keywords such as claims data, administrative data, electronic health records (EHR), electronic medical records (EMR), patient data and variations were included. Second, the applied method: studies must mention and describe the method used to analyze the data. Keywords such as data mining, sequence analysis, patient pathway analysis, clustering and others were included. Third, the presentation of the results: studies should provide a chronological sequence of care events. Combinations of keywords such as patient, care, health, utilization and trajectory, sequence, path and others were included. The keywords were kept as general as possible to keep pre-selection low. Keywords were selected, chosen and supplemented based on a preliminary literature search conducted by two authors (AF, AN). The search strategy was applied to four different databases (MEDLINE via PubMed, Scopus, EconLit, Web of Science) to cover a wide range of literature thematically and thus identify relevant studies in the fields of economic, engineering and health science. The search was limited to English language articles published between January 1, 2000 and March 16, 2021 to provide as complete an overview as possible of the methods used. As a Scoping Review is intended to provide an overview of the existing literature, the type of publication was not restricted. The full search strategy for all databases is shown in the Additional file 1, Part II.

### Study selection

All studies found as part of the search strategy were summarized and duplicates were identified and removed. Eligible articles were selected in a two-step approach by two reviewers (AF and AN). In the first step, the two reviewers examined the titles and abstracts of the identified articles for their eligibility. In the second step, the full texts of the remaining articles were checked for eligibility. To determine inter-rater agreement between the two reviewers, Cohen’s Kappa was calculated after both phases. Disagreements were resolved by discussion between the reviewers. Eligibility criteria were specified during this process. The software tools used included EndNote for initial duplicate identification, Rayyan [17] for screening stage one, Microsoft Excel for screening stage two and R to construct the resulting figures.

### Data extraction and synthesis

The categories for data extraction were determined during the second phase, incorporating the recommendations of the Joanna Briggs Institute (JBI) methodology for data extraction. For each record in the final result set, the following data were extracted: general information (authors, title, year of publication), general information about the data (types of data, setting of the data e.g. stationary or ambulatory, origin country of the data, number of included data sets), context about the study setting (disease or characteristic of the population, observation time, year of first considered data set, variables used), information about the applied methodology including used software, presentation of the results and the stated aim of the study. Results were synthesized narratively. Extracted information was compiled in Microsoft Excel. The data extraction file is shown in Additional file 2.

## Results

**Fig. 1:**
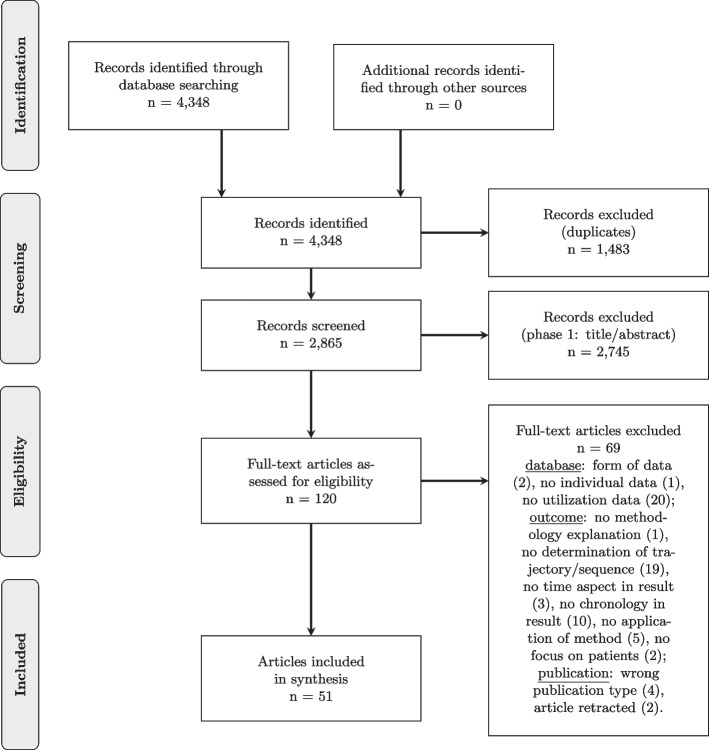
PRISMA diagram of the study selection process

The search strategy yielded 2,865 distinct articles. The titles and abstracts of 120 articles met the eligibility criteria (screening stage 1). To determine the degree of agreement between reviewers, Cohen’s Kappa was calculated. The two reviewers had high agreement in their decisions (Cohen’s kappa = 82 %). Subsequently, the full texts of the 120 studies were reviewed for eligibility. The degree of agreement between the two reviewers decisions was again very high (Cohen’s kappa = 83 %). A Cohen’s Kappa within 0.81 an 1.0 indicates an almost perfect agreement between two reviewers [18]. A total of 51 articles were identified that met the defined inclusion criteria. The Preferred Reporting Items for Scoping Reviews (PRISMA) Diagram in Fig. 1 shows the process in detail. For more detailed information on inclusion decisions for calculating Cohen’s Kappa, see Additional file 1, Part III. An overview of all included studies is presented in Table 1. In the following, a structured overview of the existing literature shall be provided. First, general characteristics of the included studies are given. Second, the first research question “What research objectives are answered in the included literature?” is presented. The main research question “What recent methods have been used to identify sequences in health data?” follows in more detail. Third, the question “How are the identified sequences represented?” is discussed. Finally, the stated aim, the method used, and the presentation of the results are placed in context. For better readability, references to the studies in interest are given in the tables, directly in the text or can be found in the Additional file 2.

**Table 1:** Overview of all includes studies including author, title, year and reference

Author	Title	Year	Ref.
Alharbi et al.	Towards Unsupervised Detection of Process Models in Healthcare	2018	[19]
Baker et al.	Process mining routinely collected electronic health records to define real-life clinical pathways during chemotherapy	2017	[20]
Bobroske et al.	The bird’ss-eye view: A data-driven approach to understanding patient journeys from claims data	2020	[21]
Cerquitelli et al.	Exploiting clustering algorithms in a multiple-level fashion: A comparative study in the medical care scenario	2016	[22]
Charles-Nelson et al.	Analysis of Trajectories of Care After Bariatric Surgery Using Data Mining Method and Health Administrative Information Systems	2020	[23]
Chen et al.	A fusion framework to extract typical treatment patterns from electronic medical records	2020	[24]
Chen et al.	A data-driven framework of typical treatment process extraction and evaluation	2018	[25]
Cheng et al.	Medical Insurance Data Mining Using SPAM Algorithm	2017	[26]
Cherrie et al.	Use of sequence analysis for classifying individual antidepressant trajectories to monitor population mental health	2020	[27]
Chiudinelli et al.	Mining post-surgical care processes in breast cancer patients	2020	[28]
Concaro et al.	Mining Health Care Administrative Data with Temporal Association Rules on Hybrid Events	2011	[29]
Dagliati et al.	Careflow Mining Techniques to Explore Type 2 Diabetes Evolution	2018	[30]
Dauxais et al.	Discriminant chronicles mining: Application to care pathways analytics	2017	[31]
Egho et al.	A contribution to the discovery of multidimensional patterns in healthcare trajectories	2014	[32]
Esmaili et al.	Multichannel micture models for time-series analysis and classification of engagement with multiple health services: An application to psychology and physiotherapy utlization patterns after traffic accidents	2021	[33]
Estiri et al.	High-throughput phenotyping with temporal sequences	2020	[34]
Han et al.	Hospitalization Pattern, Inpatient Service Utilization and Quality of Care in Patients With Alcohol Use Disorder: A Sequence Analysis of Discharge Medical Records	2020	[35]
Hilton et al.	Uncovering Longitudinal Healthcare Behaviors for Millions of Medicaid Enrollees: A Multi-State Comparison of Pediatric Asthma Utilization	2018	[36]
Honda et al.	Detection and visualization of variants in typical medical treatment sequences	2017	[37]
Hur et al.	Facilitating the Development of Deep Learning Models with Visual Analytics for Electronic Health Records	2020	[38]
Kempa-Liehr et al.	Healthcare pathway discovery and probabilistic machine learning	2020	[39]
Ku et al.	Patient pathways of tuberculosis care-seeking and treatment: an individual-level analysis of National Health Insurance data in Taiwan	2020	[40]
Lakshmanan et al.	Investigating clinical care pathways correlated with outcomes	2013	[10]
Lambert-Coté et al.	Adherence trajectories of adjuvant endocrine therapy in the five years after its initiation among women with non-metastatic breast cancer: a cohort study using administrative databases	2020	[7]
Le et al.	Analyzing Sequence Pattern Variants in Sequential Pattern Mining and Its Application to Electronic Medical Record Systems	2019	[41]
Le Meur et al.	Mining care trajectories using health administrative information systems: the use of state sequence analysis to assess disparities in prenatal care consumption	2015	[42]
Li et al.	Efficient Mining Template of predictive Temporal Clinical Event Patterns From Patient Electronic Medical Records	2019	[43]
Meng et al.	Temporal phenotyping by mining healthcare data to derive lines of therapy for cancer	2019	[44]
Najjar et al.	A two-step approach for mining patient treatment pathways in administrative healthcare databases	2018	[45]
Nuemi et al.	Classification of hospital pathways in the management of cancer: Application to lung cancer in the region of burgundy	2013	[46]
Oh et al.	Type 2 Diabetes Mellitus Trajectories and Associated Risks	2016	[47]
Ou-Yang et al.	Mining Sequential Patterns of Diseases Contracted and Medications Prescribed before the Development of Stevens-Johnson Syndrome in Taiwan	2019	[48]
Perer et al.	Mining and exploring care pathways from electronic medical records with visual analytics	2015	[49]
Pokharel et al.	Representing EHRs with Temporal Tree and Sequential Pattern Mining for Similarity Computing	2020	[50]
Rama et al.	AliClu - Temporal sequence alignment for clustering longitudinal clinical data	2019	[51]
Rao A. et al.	Sequence Analysis of Long-Term Readmissions among High-Impact Users of Cerebrovascular Patients	2017	[52]
Rao A. et al.	Common Sequences of Emergency Readmissions among High-Impact Users following AAA Repair	2018	[53]
Rao G. et al.	Identifying, Analyzing, and Visualizing Diagnostic Paths for Patients with Nonspecific Abdominal Pain	2018	[54]
Righolt et al.	Classification of drug use patterns	2020	[55]
Roux et al.	Use of state sequence analysis for care pathway analysis: The example of multiple sclerosis	2018	[6]
Solomon et al.	The sequence of disease-modifying anti-rheumatic drugs: pathways to and predictors of tocilizumab monotherapy	2020	[56]
Sun et al.	Mining information dependency in outpatient encounters for chronic disease care	2013	[57]
Vanasse et al.	Healthcare utilization after a first hospitalization for COPD: a new approach of State Sequence Analysis based on the ?6W? multidimensional model of care trajectories	2020	[5]
Vogt et al.	Applying sequence clustering techniques to explore practice-based ambulatory care pathways in insurance claims data	2017	[58]
Wang et al.	A framework for mining signatures from event sequences and its applications in healthcare data	2013	[59]
Wright et al.	The use of sequential pattern mining to predict next prescribed medications	2005	[60]
Yan et al.	Learning Clinical Workflows to Identify Subgroups of Heart Failure Patients	2016	[8]
Zhang et al.	On Learning and Visualizing Practice-based Clinical Pathways for Chronic Kidney Disease	2014	[61]
Zhang et al.	Innovations in Chronic Care Delivery Using Data-Driven Clinical Pathways	2015	[62]
Zhang et al.	On clinical pathway discovery from electronic health record data	2015	[9]
Zhang et al.	Paving the COWpath: Learning and visualizing clinical pathways from electronic health record data	2015	[66]

### General characteristics of identified studies

Most articles were published in the last five years (35, 68 %) (2017-2021). Many articles were also published in the previous five years (14, 27 %) (2012-2016). Only two articles (4 %) were published in the entire preceding period under consideration (2000-2011).

#### Data source and data information

Administrative data were used in the majority of the included studies (22, 43 %) to construct the sequences, followed by Electronic Health Record Data (EHR, 11, 22 %) and Electronic Medical Data (EMR, 9, 18 %). The different data types are briefly introduced. Administrative data or claims data are routinely collected data that include diagnoses, treatments, medications, procedures and outcomes given. They are submitted to payers and used for billing and administrative purposes [63, 64]. EHR are continuously captured and maintained by the provider and contain a patient’s medical history in electronic form. It includes all relevant clinical patient information such as demographic data, medication, vital signs, lab data, medical history, health issues, immunizations and radiology reports [65]. EMR are collected by individual providers and include, among others, vital signs, lab results, non-prescriptive drugs, patient surveys, patient habits (alcohol use, smoking) and information recorded by members of the disease management [63]. The data used by a further nine studies (9, 18 %) were not assigned to any of these types. Some studies (3, 6 %) use data from the publicly available U.S. database MIMIC III (Medical Information Mart for Intensive Care III). Other studies use hospital data, outpatient encounter data or data of accidents and the utilization of psychologist and physiotherapists. Considering the sector in which the data arose, most articles (11, 22 %) focus on the hospital sector, some (4, 8 %) focus on the ambulatory/outpatient sector, and the remaining articles (36, 71 %) do not specify the sector. The country of origin varies. Most articles (17, 33 %) analyze data from the USA. French data are analyzed in seven studies (14 %), and data from United Kingdom, Italy and China are each considered in four studies (8 %). Three articles (6 %) examine Canadian data. Japanese and Taiwanese data are considered in two studies (4 %). A single study was found for Australia (1, 2 %), Germany (1, 2 %), New Zealand (1, 2 %) and Portugal (1, 2 %). In some cases (4, 8 %) the country of origin was not clearly defined. Looking at the entire list of authors of the articles included in each case, several overlaps in authorship were identified. This includes two articles considering Japanese data [37, 41], four articles using Italian and French data [28–31], two articles using British data [52, 53], four studies using mainly Chinese data [9, 61, 62, 66], two more articles using Chinese data [24, 25] and another five articles using data from various countries [10, 37, 43, 49, 59]. The bibliography contains more information about the authors of the studies.

#### Population and sample size

In the selected studies, many different criteria were used to select the cohort of patients under consideration. To provide a unified grouping for comparison in this scoping review, patients’ characteristics are classified using the categories of International Statistical Classification of Diseases and Related Health Problems 10th Revision (ICD-10) coding [67]. The ICD-10 chapters of patient characteristics of the included studies are displayed in Table 2. Most studies analyze the health care data of patients with diseases of the circulatory system (ICD-10 chapter 9; 10, 20 %), such as heart diseases (6, 12 %) and stroke (2, 4 %), or data from patients with endocrine, nutritional and metabolic diseases (ICD-10 chapter 4; 10, 20 %). Studies of patients with endocrine, nutritional and metabolic diseases focus largely on patients with a type of diabetes as main criterion (9, 18 %) and one study included patients with obesity after a bariatric surgery (1, 2 %). Eight studies (16 %) analyzed the data of patients with neoplasm. The treatment of lung cancer (3, 6 %), breast cancer (2, 4 %), breast or colorectal cancer (1, 2 %) and the transurethral resection of a bladder tumor (2, 4 %) was also studied. Some studies (4, 8 %) by concurring authors analyzed data of patients with chronic kidney disease which is a disease of the genitourinary system (ICD-10 chapter 14). Two publications considered data sets of rheumatoid arthritis patients (2, 4 %) and two publications pregnant women (2, 4 %). Other criteria for inclusion of individual data sets included the analysis of surgical pathways and a selection of different diseases. The observation period considered in the included articles range from 14 days to 24 years. Most studies considered time horizons between one year and 10 years (24, 47 %), some studies analyzed longer observation periods (10, 20 %) and a smaller number examined shorter observation periods (5, 10 %). Eleven studies (22 %) did not specify the length of the time horizon. The date of the first data collection of all considered studies was between 1991 and 2005. The median year of the first data collection was 2009. According to the definition of eligibility, the included articles used individual patient data. The size of the patient population in the different articles varies. In a few articles with a comparatively small population, fewer than 500 patients were studied (8, 16 %). Most articles analyzed between 500 and 2,000 (14, 27 %) and between 2,000 and 20,000 (19, 37 %) patients. Other studies looked at between 20,000 and 200,000 patients (9, 18 %), with only one study analyzing over 200,000 patients (1, 2 %). The median number of patients considered was 2,581.

**Table 2:** Diseases of the patients in the included studies. Classification according to the International Statistical Classification of Diseases and Related Health Problems 10th Revision

Chapter	Description of Chapter according to ICD-10 classification	N (%)	Ref.
1	Certain infectious and parasitic diseases	2 (4 %)	[40, 50]
2	Neoplasms	8 (16 %)	[7, 20, 28, 32, 37, 41, 44, 46]
4	Endocrine, nutritional and metabolic diseases	10 (20 %)	[22, 23, 29, 30, 47, 49, 55, 57, 59, 60]
5	Mental, Behavioral and Neurodevelopmental disorders	2 (4 %)	[27, 35]
6	Diseases of the nervous system	2 (4 %)	[6, 31]
9	Diseases of the circulatory system	10 (20 %)	[8, 10, 24, 26, 38, 43, 45, 52, 53, 58]
10	Diseases of the respiratory system	2 (4 %)	[5, 36]
12	Diseases of the skin and subcutaneous tissue	1 (2 %)	[48]
13	Diseases of the musculoskeletal system and connective tissue	3 (6 %)	[21, 51, 56]
14	Diseases of the genitourinary system	4 (16 %)	[9, 61, 62, 66]
15	Pregnancy, childbirth and the puerperium	2 (4 %)	[26, 42]
18	Symptoms, signs and abnormal clinical and laboratory findings, not elsewhere classified	2 (4 %)	[19, 54]
20	External causes of morbidity	1 (1 %)	[33]
–	other criterion for inclusion of individual data sets	2 (4 %)	[34, 39]

#### Use of variables

The included studies considered different kinds of utilization events to identify sequences of care. The different events, used as variables in the technical models, are summarized by grouping them into types of events. Some studies used multiple types of (utilization) events, which is why the total exceeds 100%. Medical and diagnostic procedures were often used (28, 55 %) as utilization events. In particular, lab tests [10, 21, 38, 43, 57], medical treatments [6, 23, 24, 33, 37, 40, 41], examinations [22, 54, 58] and surgery [28] were considered. Prescription medication was frequently considered (21, 41 %) including the drug class [60], drug purchases [30] or medication use [7]. Further studies use prescription data (11, 22 %) as care events, which can include various events related to medication and treatment. In nearly half of the included records (23, 45 %), visits to medical institutions were analyzed, including consultations in general [5, 45] as well as inpatient visits [6, 36, 46, 48, 52, 53, 61], outpatient consultations [6, 36, 45, 48, 55, 59], hospitalizations [30, 31, 50] and the consultation of specialist physicians [5, 26, 58]. The studies included sometimes considered additional aspects of health care. Although we do not focus on these kind of events in this review, these are described for completeness. Several articles analyzed the diagnoses given to patients (21, 41 %), data on health status and results of examinations (9, 18 %), as well as other information such as medical records or billing data (5, 10 %).

### What research questions are stated in the identified literature?

This subsection gives an overview of the research questions stated in the included studies. Later, the stated research aims are set in relation to the method applied. In this way, it is hoped that readers of the present scoping review will be better able to classify their own research aims and to identify about possible methods. Again, the stated goals were categorized based on a classification that emerged from the review of the literature. However, the resulting groups are not mutually exclusive, which is why some studies were assigned to more than one objective. The most frequently cited research objectives were the identification and presentation of care trajectories (28), followed by the development and presentation of a (new) method (27) and the extraction of care patterns (20). A small number of studies aimed to identify phenotypes (9) or to predict specific events or health states (11). As already mentioned in the background section, we distinguish between care trajectories (care pathways) as a sequence of utilization events spanning an entire observation period and patterns of care that represent common subsequences within this period. Both concepts take the chronological order of utilization events into account. The distinction is exemplified by the following example: Consider the utilization of physicians (general practitioner GP, specialist practitioner SP) and let the utilization sequences of two patients with heart failure be GP-SP-GP-GP and SP-GP-GP-GP for a defined time horizon. With the aim of identifying the same trajectories of physician utilization in the given example, no match would occur. If one were to look for patterns of utilization and a subsequence of two consecutive events is defined to be sufficiently long, the subsequence GP-GP would be a matching pattern of utilization between the two care sequences. This information can be used to look more closely at the content objectives of the studies. For example, the literature on identifying care trajectories considered in this review aims to analyze the complexity and heterogeneity of a care pathway [40], to quantify complications in treatments [23], to discover temporal hospitalization admissions [38], or to model the impact of a specific patient self-test service in patients undergoing chemotherapy treatment [20, p. 33]. Occasionally, the trajectories were constructed for use in a prediction model. In such cases, the aims included the prediction of unplanned cardiac surgeries that could be integrated in a system for medical staff to test clinical hypotheses [38], the discovery of factors associated with the trajectories [42], and the identification of features related to the trajectories that either affect patient recovery time [39] or are associated with a monotherapy using the drug tocilizumab [56]. Other studies were designed to facilitate phenotyping, the identification of groups of patients with characteristic conditions or outcomes [68]. In particular, these studies aimed either to find a group of patients with similar treatment patterns and characterize a population most at risk from a progressing disease (e.g. mental health) [27], to classify patient groups associated with utilization patterns [33], to find groups of patients with diabetes who had similar drug use (metformin use) [55], or to identify therapy profiles [51]. As noted earlier, the categorization of targets is not unique, and the results of studies looking for trajectories, patterns, or groups of patients with matching characteristics will sometimes have very similar results. The literature reference for each stated research objective can be found in Table 3. The full statements of the stated objectives are documented in Additional file 2.

**Table 3:** Aggregated results of the included studies. Multiple entries of the same article are possible in the categories of the specified aim and the presentation of results

**Stated aim in article**	**N**	**Publications**	
Proposal of method	27	[6, 8–10, 20, 21, 24, 29, 31, 34, 36–38, 40, 41, 43–47, 49, 51, 55, 57, 59, 62, 66]	
Trajectory	28	[5–10, 20, 21, 23, 25, 27, 35, 36, 38–40, 42, 44–47, 54–56, 58, 61, 62, 66]	
Patterns	20	[19, 24, 26, 28–33, 37, 41, 43, 48–50, 52, 53, 57, 59, 60]	
Phenotyping	9	[22, 27, 28, 33, 34, 44, 46, 51, 55]	
Prediction	11	[7, 10, 27, 31, 35, 38, 39, 47, 56, 58, 60]	
**Stated method in article**	**N (%)**	**Publications**	
**Clustering**	**16 (31 %)**	[5–7, 21–23, 27, 35, 42, 44–46, 51, 55, 58, 61]	
Hierarchical	7	[5, 6, 27, 35, 42, 51, 61]	
Partitioning	5	[21, 44, 46, 55, 58]	
Other Clustering	4	[7, 22, 23, 45]	
**Pattern Mining (PM)**	**16 (31 %)**	[10, 26, 28, 30–32, 34, 37, 38, 41, 43, 48–50, 57, 60]	
PM + Clustering	3	[10, 30, 37]	
**Markov Model**	**10 (20 %)**	[9, 19, 20, 24, 25, 33, 36, 56, 62, 66]	
MM + Clustering	7	[9, 24, 25, 33, 36, 62, 66]	
**Other**	**9 (18 %)**	[8, 29, 39, 40, 47, 52–54, 59]	
**Presentation of results**	**N**	**Publications**	
**Visualization**	**36**		
Trajectory	28	[5–10, 21, 23, 25, 27, 28, 30, 33, 35–40, 42, 44, 45, 54–56, 58, 61, 66]	
Patterns	8	[19, 24, 31, 34, 41, 49, 51, 59]	
*Weighted*	8 / 4	Trajectory: [23, 25, 33, 36, 39, 45, 54, 56]	Patterns: [19, 24, 31, 51]
*Sankey*	5 / 1	Trajectory: [21, 38, 40, 44, 49]	Patterns: [49]
*Timeline*	10 / 1	Trajectory: [5–7, 27, 35, 40, 42, 55, 56, 58]	Patterns: [24]
**Tabular**	**22**		
Trajectory	5	[20, 31, 46, 47, 62]	
Patterns	17	[10, 22, 26, 29, 32, 37, 38, 43, 44, 46, 48–50, 52, 53, 57, 60]	
*Weighted*	2/14	Trajectory: [20, 47]	Patterns: [10, 22, 29, 32, 37, 38, 43, 44, 46, 48, 50, 52, 53, 60]

### What recent methods have been used to identify sequences of health care utilization?

The process of identifying sequences in data sets usually consists of several stages. It is therefore not always easy to classify the methodology used in a study. This review takes the approach of sorting articles with respect to the method that is the focus of sequence identification. The included studies applied methods of machine learning such as clustering algorithms (16, 31 %), different pattern mining algorithms (16, 31 %), (hidden) Markov models (10, 20 %), and other probability based methods e.g. constrained probability and further methods (9, 18 %). Preliminary methods like data preparation and post-processing methods were not in focus but were considered partially in the description of the techniques, especially if the pre- or post-processing involved clustering methods.

The main features of the most commonly used methodological approaches are described. A summary of the extracted results and the related studies is presented in Table 3. Occasionally, the authors indicated making associations or predictions about the occurrence of future events. The stated goal of establishing relationships to future events is indicated in Table 3. References to association methods used are made in the following sections. Due to the different focus of the study, prediction methods are not explained in detail.

#### Cluster analysis

Clustering algorithms are used to group objects into subsets according to a defined measure of similarity. To do this, measures of similarity or relation between the objects are considered. Objects within a cluster are therefore more similar to each other than they are to objects of different clusters [69, p. 501].

A total of 16 (31 %) studies focussed on clustering algorithms to identify sequences of care utilization. Most (13) aimed to find trajectories of care or common patient flows in the data, indicating the consideration of the entire time horizon. Thus, groups of patients with the same sequences of care were formed. To apply a clustering method, it is necessary to clearly define the possible utilization events and the temporal section within the considered time horizon. These decisions can affect the results. The methodological approach allows to extract clear and comparable care sequences, making it possible to track individual care sequences. Furthermore, many software tools and programming languages have mature clustering libraries, simplifying the implementation. This will be explained in more detail later. Clustering is also applied to group patients according to specific characteristics, or to group identified pathways retrospectively as a form of data pre- or postprocessing. In the included literature, twelve (24 %) more studies apply clustering methods to do this. Clustering requires the definition of a similarity (or dissimilarity) measure as well as an algorithm to combine objects based on their calculated similarities. Unfortunately, not all studies explicitly stated which measure of similarity they applied. The use of Optimal Matching to calculate the similarity or dissimilarity of the sequences was documented in five studies [5, 6, 27, 35, 42]. The idea of Optimal Matching is to calculate fictional costs pairwise between sequences, i.e. the costs to transfer one sequence into another sequence. Other approaches used to compute dissimilarities are the Needleman-Wunsch algorithm (1) [51], the Longest Common Subsequence (LCS) (2) [58, 61], the Levenshtein edit distance (1) [21], a cosine distance measure (1) [22] and the Euclidean distance (1) [44]. There are no discernible, pre-defined criteria (for example, events used) to explain why researchers decided to use one particular similarity measure over another. It is possible that some researchers applied several measures, reporting only the best-performing measure. After calculating the pairwise similarity of the sequences contained in the dataset, clustering is performed to group objects (sequences) with greater similarity. Different types of clustering algorithms exist, including hierarchical and partitioning algorithms. Hierarchical algorithms were often applied (7, 14 %). Using agglomerative hierarchical clustering methods, each sequence starts out as its own cluster. These clusters are combined iteratively, increasing the number of objects per cluster and reducing the number of clusters. The number of clusters is therefore not defined in advance. Ward’s Linkage is a method that considers the least variance increase when merging clusters [70], and was used in six studies [5, 6, 27, 35, 42, 51].

Partitioning methods represent a further type of clustering algorithms. The number of clusters is determined in advance, which can be challenging to define. Objects are assigned initially to cluster centers and reassigned iteratively to minimize an objective function. Partitioning methods were applied less frequently in the identified literature (5, 10 %) [21, 44, 46, 55, 58]. In particular, four studies [21, 44, 46, 55] stated that the k-means algorithm was used. This was occasionally supplemented by considering specific centres (bayrcentres) [46] or by conducting several iterations of k-means using ensemble clustering [21]. K-medoid, another kind of partitioning clustering method, was applied once [58]. Several studies used a combination of different methods, which are not explained in more detail, but stated for completeness (4, 8%) [7, 22, 23, 45]. For example, one study used a group-based trajectory modeling approach to clustering [7]. The method itself was merely stated, with the authors referring to Nagin et al. [71], which explains that the group-based trajectory model uses a probability based method for clustering. A formal concept analysis [23] or the combination of a variety of existing clustering algorithm were used only once [45]. Among others, k-prototype algorithms, average linkage criterion and k-means were applied. Finally, a framework was proposed which combined a variety of multiple-level clustering algorithms and a maximal sequential pattern miner programmed in Java [22]. This applied several density-based (Multiple-Level DBSCAN), hierarchical (bisecting k-means) and partitioning (bisecting k-medoids) clustering methods.

Often clustering results were used as a starting point for further analysis. Some of the included studies stated that the aim was prediction, association or relation with health states. Logistic regression models were applied several times. Multilevel logistic regression was used to determine associations between the characteristics of considered patients and the assignment to identified clusters of care trajectories [27]. Similarly, multinomial logistic regression was applied to identify healthcare-related and sociodemographic characteristics associated with trajectories [7] and a logistic regression was conducted to find “most effective sequences for avoiding hospitalizations” [58, p. 214]. Also, various statistics as the Kruskal-Wallis test, Mann-Whitney U test, Chi-square test and Bonferroni correction were used to compare constructed sequences of hospitalizations to determine associations with care utilization [35].

Several articles (12, 24 %) used clustering or grouping as a way to prepare data. For example to aggregate several parameters [39] or patients [8], to remove outliers [10], or to cluster patients’ trajectories before applying further methods [9, 62, 66]. On the other hand, clustering methods were used as a kind of postprocessing to aggregate the obtained results (6, 12 %) [24, 25, 30, 33, 36, 49].

#### Pattern mining algorithms

Pattern mining aims to find elements or states in a database that are related in some way [72, p. 1]. The focus of this method is to identify frequently occurring sequences (patterns) that consist initially of two successive events. In a dataset of care trajectories, a pattern is identified in the following way: a transition from one care event to another care event is considered as frequent if the rate at which the transition occurs in all sequences of the dataset is greater than a defined minimum support threshold [72]. In this case the specific transition is seen as a frequent pattern. This leads to the conclusion that pattern mining algorithms only detect frequent subsequences of care trajectories at first and do not construct an event flow over the complete time horizon. One example for the use of such a method is the detection of frequent changes in medication use by considering medication prescriptions as care events. The ability to modify the minimum support threshold, what leads to different strengths of the identified patterns, can be advantageous.

Within the identified literature, 16 (31 %) articles documented the use of pattern mining. Most of the used methods were variations of existing methods, including the sequential pattern mining algorithm using a bitmap representation (SPAM), sequential pattern discovery using equivalent classes (SPADE), PrefixSpan and a closed sequential pattern algorithm (CloSpan). Sequential pattern mining methods identify patterns which take the chronology of the considered events into account, which was a component of the defined eligibility criteria for the present review. A small number of studies [10, 26, 43, 49] based their method on the SPAM algorithm, a method developed by Ayres et al. [73]. Changes and extensions were made within the included studies, including for example the consideration of the occurrence of several events at the same time, or using Bag-of-Pattern (BoP) vectors to represent each patient’s path [10]. Further extensions of SPAM are the ability to consider temporal information associated with events [43] and to incorporate inter-event duration constraints and the association with a positive or negative health outcome of each patient [49]. A variation of SPADE, called cSPADE, was introduced by Zaki et al. [74] and was used once in the identified literature [60]. Zaki et al. extended SPADE to allow the addition of constraints on the length and time period of the sequences. The authors borrowed from the approach taken in prediction, applying 10-fold cross validation to improve the quality of the results. This method was applied to identify medication prescription patterns, allowing prediction of the next prescribed medication. Further studies [37, 41] applied a T-PrefixSpan algorithm, which mines time-interval sequential patterns and can represent variants in the patterns. This can be helpful if slight differences in the sequences are to be accounted for in the results explicitly. Other sequential pattern mining algorithms used were Clospan, a closed sequential pattern mining algorithm [38] and MMISP, a multidimensional mining algorithm [32]. Careflow Mining approaches were also used occasionally [28, 30]. These combine sequential pattern mining and temporal data mining methods following the approach presented in Dagliati et al. [75]. Other methods used were a combination of a sequential pattern mining algorithm with gap constraint and a temporal tree technique [50], a generalized sequential pattern algorithm [48], a transitive sequential pattern mining algorithm [34], an algorithm that defines a rolling time window to consider the temporal aspect of the considered events [57]  and a temporal pattern mining algorithm [31]. Again, pattern mining results were sometimes used for subsequent analysis. Some studies set out to associate these patterns with health states. The prediction of unplanned surgeries was performed by applying a deep learning method, namely a Long Short-Term Memory (LSTM) attention model [38]. The association between identified hospitalizations and drug switches was identified by means of direction cosine matrix (DCM) algorithms [31]. Similarly, the prediction of the next prescribed drug was drawn from pattern mining results [60]. Finally, one study calculated the correlation of frequent patterns with patient outcomes [10].

In conclusion, some articles can be grouped according to a common characteristic. Namely, some studies that apply sequential pattern analysis can be assigned to a group of articles which focus on the time aspect in a more detailed way [37, 43, 50]. Several articles base their technique on SPAM, as constructed by Ayres et al. [10, 26, 43, 49], some studies use methods based on T-PrefixSpan [37, 41] and others using the CloSpan algorithm [32, 38].

#### Markov model

The principle of the Markov model is to describe health, disease, and treatment as a sequence of states, considering the transition probabilities from one state to another. A basic assumption of Markov chains is that the current state depends only on the value of the previous state [76]. Within the identified literature, 10 (20 %) studies applied Markov chains. Sequences and patterns were described by calculating the probability of a transition from a given event to a subsequent event. Again, some of the included studies combined the method with additional techniques. For example, pathways were first simplified and Markov chains applied to calculate transitions subsequently [20], or discrete-state Markov models were calculated and logistic regression applied to determine the longitudinal factors associated with medication use [56]. Additionally, one study reported the application of a hidden Markov model in combination with a Viterbi algorithm [36]. Hidden Markov models are based on classical Markov chains but differ in the specification of the transition probability matrix [76]. To indicate the resulting transition probabilities with respect to a patient cohort, the remaining studies applied clustering (7). In some of these cases (3), clustering of the individual patients’ sequences was performed [9, 61, 62]. In particular, hierarchical clustering methods incorporating the Longest Common Subsequence distance measure and Ward’s method were applied and the Markov chains calculated per cluster. The remaining articles (4) applying clustering after constructing Markov chains differ in both the distance measure and the clustering method [24, 25, 33, 36].

#### Other methods

Another nine articles applied methods that do not fit into one of the defined groups. This included statistical techniques applying conditional probability (2) [47, 54], a probability based patient pathway analysis (1) [40], and a presentation of a stochastic optimization scheme to perform group-specific temporal event signatures based on convolutional non-negative matrix factorization (cNMF) with added sparsity regularization (1) [59]. State sequence analysis, a sequence cluster method originally from the social sciences for the analysis of life histories, was applied in two studies using predefined patient populations [52, 53]. An algorithm to extract temporal association rules (TAR) to identify frequent patterns was found in one study [29]. One study clustered patient groups using the similarity of refined sequences with k-means hierarchical clustering and applied an inductive mining algorithm to derive the sequences as it is implemented in ProM [8]. The authors of another study stated use of ProM [77] software to extract pathway variants from patient traces [39].

Again, the association between the sequence clusters and heath states was analyzed. Using ProM, further probabilistic programming was performed to investigate the characteristics of the trajectories that affect patient recovery time [39]. Furthermore, a multivariable logistic regression model was chosen to calculate the influence of the trajectories and comorbidities on the risk of progression of the disease diabetes [47].

#### Software

In the following, reference is made to software packages that were frequently used. Several studies (6, 12 %) made use of the TraMineR Package for R, with its support for state sequence analyses to facilitate clustering [5, 6, 27, 35, 42, 58]. Similarity measures and clustering algorithm are provided by TraMineR but can be modified to meet the needs of individual projects [78]. Some studies (2) conducted a state sequence analysis in TraMineR on grouped patients without explaining the procedure in more detail [52, 53]. A similar tool for the identification of sequences and for patient pathway analyses is the Java tool ProM. ProM is a framework supporting different process mining techniques [77] and is applied on health data by two of the included studies [8, 39]. One study uses Python to conduct a patient pathway analysis [40]. Finally, a commercial platform called business process insight (BPI) is presented which “provides an event-driven process-aware analytics toolset” [10, p. 323] to perform clinical care pathway analysis.

### Which presentation of results was chosen?

As required by the defined eligibility criteria, all included studies presented their results visually or in tabular form, with the temporal order considered in all result presentations. Table 3 summarizes the choice of presentation. We again differentiate between care trajectories and common subsequences and understand a trajectory as a sequence of care events spanning the whole observation period, whereas common sequences that cover only part of the defined observation period are called patterns throughout this paper. The resulting care pathways are usually presented visually by using a modified flow chart diagram. Most studies that claimed to identify care trajectories or phenotypes of care pathways have taken this approach. Methods such as Markov models [36, 56], process mining [25] and formal concept analysis [23] yield paths with numerical weights for the transitions. Some visual representations included this weighting in the form of a numerical indication or encoded via the thickness of the transition symbols. This can be helpful in quantifying outcome events as either postoperative complications or unexpected events [23]. Sankey diagrams are a flow chart variant which visualize the patient streams over a defined time period and can consider fixed time segmentation. They are frequently applied to represent trajectories [21, 38, 40, 44, 49]. As with other representations considering the transition of states, it provides a clear overview of frequent transitions within a fixed time period. One advantage of Sankey diagrams is that they represent frequent processes occuring within a complete trajectory. Unfortunately, they do not allow individual paths to be traced, but can be augmented to show a direct relation to a defined timeline, as found in one of the included studies [40]. In total, eleven (21 %) studies additionally considered the temporal dimension of the sequence in more detail and presented the resulting (sub-)sequence in relation to a timeline. They mostly focussed on the use of medication and outpatient treatment. The research aims of the studies that considered the temporal aspect in this way were the prescription of metformin for diabetes patients [55], medication use for breast cancer patients [7], changes in prescriptions for mental health [27], the identification of care utilization groups in multiple sclerosis [6] or heart failure [58], care seeking patterns in tuberculosis [40], and prenatal treatment [42]. One of the studies presented the most frequent utilization pathways [58]. This visualization method made it possible to trace the individual path of the patients considered in those pathways and can be advantageous if information on the explicit care sequence of individuals is needed.

The stated aims for generating common subsequences were to select patients who experienced specific paths that were of interest to medical staff [38], to find patterns of medical orders [37] or association rules for the utilization behaviour of diabetes patients [29]. Markov models [40] and pattern mining algorithms [49] provide insights on patient flows in a similar way to Sankey diagrams.

In conclusion, identified trajectories were more likely to be visualized (28, patterns only 8) and patterns were more likely to be presented as a table (17, trajectories only 5). As patterns can consist of a pair of two consecutive events, there seems to be less utility in visualizing such a short sequence. The visualized (sub-)sequences can be divided into visualization types that allow the paths of individual patients to be tracked, and those that show the relative frequency of different transitions along the time axis.

### Relations between stated objectives, methods used and presentation of results

**Fig. 2:**
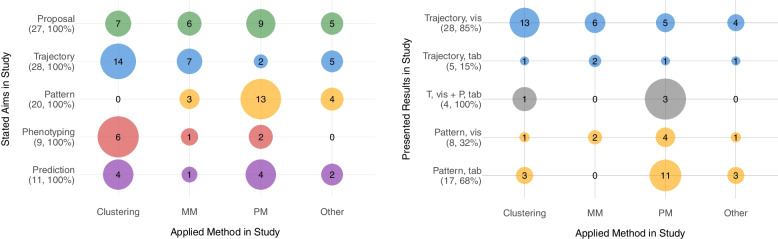
Relation of stated aim, used method and presentation of result. The figure on the left shows the proportion per specified aim using a particular method in the study (size of the same color bubbles). The figure on the right shows the proportion per specified outcome using a particular method in the study. The size of the bubbles represents the proportion [in percent] within the specified aims (left graph) and the proportion within a parent result type (right graph). The values within the graphic indicate the number of studies which meet the given characteristics on the respective axes. Applied methods include Clustering, Markov Models (MM), Pattern Mining (PM) and other methods (Other). Presented results include Trajectories (Trajectory, T), Patterns (Patterns, P) or both (T+P) in a tabular (tab) or visualized (viz) way

The information extracted on the stated aim, the applied methods and the presentation of the results are now put in relation to each other to enable readers to classify their own research project and select the appropriate methodology. Figure 2 presents two bubble charts to assist in this task. On the left side of the figure, the stated aim of the included articles (y-axis) is shown in relation to the method category used (x-axis). The total number of included studies following each aim is annotated in the labels of the y-axis. The bubbles break down each objective into the methodologies applied, the size of the bubbles shows the proportion of times each method was used to achieve the respective goal, with the number inside the bubble giving the absolute number of studies. The bubble sizes serve to compare the frequency of methods used to achieve the same aim. The graph shows that studies seeking to propose a new method use all of the specified methods with similar frequency (5 - 9, 19 % - 33 %). Further, it indicates that most articles which state to identify trajectories in the data sets apply clustering algorithms (14, 50 %) to do so, with the next most common method being Markov models (7, 25 %). Looking at the methods chosen in the studies that aim to find patterns in the data, the ordering of the methodologies is reversed, with pattern mining methods most frequently applied (13, 65 %), followed by Markov models (3, 15 %) and other methods (4, 20 %). In studies aiming to predict, clustering (4, 31 %) and pattern mining methods (4, 31 %) are applied with similar frequency, and Markov models found infrequently (1, 8 %).

Studies which focus on finding phenotypes in the data sets mostly apply clustering (4, 36 %) and pattern mining algorithms (4, 36 %), with Markov models again found infrequently (1, 9 %). Further details on the objectives and methods mentioned can be found in the respective sections above.

On the right hand side of the Fig. 2, the results presented in the study are related to the methodology employed. A distinction is made between studies that display trajectories (Trajectory), trajectories and patterns (T+P), and patterns (Pattern). These are further subdivided into those that displayed visualized results (vis) as opposed to tabular results (tab). Within a parent result type (Trajectory, T+P, Pattern), the percentages represented by the bubble size sum to 100%. The graph shows that studies presenting a visualized trajectory mostly apply a clustering method (13, 39 %), followed by Markov models (6, 18 %) and pattern mining (5, 15 %). A tabular presentation of trajectories is rather seldom. Studies presenting both trajectories and patterns (T+P) use pattern mining (3, 75 %) or clustering (1, 25 %). In studies visualizing patterns of care (Pattern, vis), the various methods were used with similar frequency. Whereas pattern mining was used a little more often (4, 16 %). Pattern mining methods are most often applied to present common subsequences in a tabular (11, 44 %) followed by clustering methods (3, 12 %) and other methods (3, 12 %).

## Discussion

This scoping review summarizes the range of methods that are used to identify sequences in health data. The scoping review illustrates that the studies can be divided into studies that seek to identify patterns in data and studies that map trajectories. This distinction is also central to the choice of method, with a clear difference seen between the two groups. Clustering was the predominant method used for the identification of trajectories. Clustering methods allow understanding to be gained on complete care pathways, and the researcher can choose whether to obtain results that explicitly show individual pathways or to obtain an overview of commonly used care streams without results at an individual-level. Clustering requires that all eligible health care events be defined in advance, which can be considered a limitation of the method. Various similarity measures have been applied as basis for clustering and there is no clear indication as to which measure should be used for a particular situation. For the identification of patterns, variants of sequential pattern recognition methods were most commonly used. In the present review, patterns are understood as frequent segments of the complete care sequence which allow for information about common combinations of care events. This can for example be helpful in finding undesirable care transitions. Variations of Markov models were also used in both cases. Regardless of the main method applied, clustering methods have also been used to preprocess the database or postprocess the results. Most studies used a combination of different techniques to prepare and analyze the data, meaning that distinctions between the methods are not always clear-cut. Additionally, the studies explain the methodology in widely varying detail, and often lack a rationale for the choice of methodological details, such as the choice of similarity measure for clustering. The elaboration of the method types listed in this review and the assignment of studies were undertaken to the best of our knowledge and represent a central result of this review. We recommend that authors should describe their methodology as transparently as possible. Future articles should aim to present the methods used as completely as possible and in a structured manner. This should include both the reasons for the chosen techniques and the limitations of the method (e.g. type of data set, sizes of data sets, computing time and others). A full description of the dataset should also be provided. In addition to the very heterogeneous descriptions of the methodology used, the wide heterogeneity in the exact implementation of the methods is conspicuous and indicates that there is still a need for discussion and research in order to specify the best possible algorithmic details. Further research should apply the identified methods to comparable datasets, evaluate them in terms of their performance and against established quality criteria to create a quantitative comparison of methods. We recommend a methodological study that provides recommendations and evaluations of the methods found in this scoping review for future research.

Looking at previously published literature on reviews of the topic area of treatment processes in health data, it can be seen that there are clear limitations. Reviews either included only a very limited range of methods [11–13] or cover an outdated time period without presenting the method used and its application [14]. Considering the number of published studies with such data analyses, it is noticable that the number of studies analyzing health data to identify care sequences has increased strongly, especially in the last five years. The present review considers all algorithm-based methods published in studies between 2000 and 2021 that met the inclusion criteria. To achieve a clear literature search and minimize bias, we adhered to the suggested procedure of the JBI-methodology and the ScR checklist and defined clear inclusion criteria. These require studies to use health data, specify the method used and present results and led to the exclusion of purely technical approaches and approaches that might be applicable to health data but currently are not. This can also be seen as a limitation but was made to ensure the suitability of the application to health data and to ensure a certain comparability of the methods. We break the methods of the included studies down to the basic method applied and provide readers with a structured overview. Since the use of different techniques and the varying level of detail in the method descriptions make it difficult to quickly understand the procedure and classify the content of the method, this provides substantial benefit for researchers. Classifying the identified methods in relation to the stated research objective and the resulting outcomes allows new researchers to make an informed decision when choosing methods. In summary, the analysis of individual health care data and the construction of health care pathways and subsequences holds great potential for understanding real-world care processes in order to uncover deficits and gaps in health care delivery. The knowledge obtained should be used to validate and discuss guidelines, and to inform recommendations for the improvement of care processes the future optimization of health care delivery.

## Conclusions

In conclusion, this scoping review presents the variety of methods applied to identify care sequences in health data. The stated target questions, the method used and the results obtained are set in relation to each other. The methods used are divided into those methods aiming at extracting patterns and those aiming to identify full care trajectories. Patterns are mostly identified by sequential pattern recognition methods, whereas cluster methods are mostly used to extract trajectories of care. Using this scoping review, readers can inform themselves about the current state of the methodology and make an informed decision when choosing their own method.

### Supplementary information

**Additional file 1.** Search Strategy, Inclusion and Exclusion criteria, Cohen’s Kappa. Part I contains the defined inclusion and exclusion criteria against which the data sets are reviewed for eligibility. Part II contains the structure of the search strategy and the search strategies for each database. Part III contains data to calculate Cohen’s Kappa as measure for agreement in the screening process.

**Additional file 2.** Data extraction. Part I includes extracted general information, Data Source, Setting and Population of the included articles, Part II includes the used events for constructing the sequences per included study and Part III includes information about the applied methodology, the Software, presentation of results and the stated aim of the study.

**Additional file 3.** PRISMA ScR Checklist. Completed PRISMA-ScR checklist for this scoping review. An appendix contains supplementary information that is not an essential part of the text itself but which may be helpful in providing a more comprehensive understanding of the research problem or it is information that is too cumbersome to be included in the body of the paper.

## Data Availability

The material used is available from the author upon request.

## Abbreviations

PRISMA-ScR: Preferred Reporting Items for Systematic Reviews Extension for Scoping Reviews
JBI: Joanna Briggs Institute
EHR: Electronic health records
EMR: Electronic medical records
ICD-10: International Classification of Disease and Related Health Problems 10th Revision
PM: Pattern Mining
MM: Markov Model
GP: General practitioner
SP: Special practitioner

## References

[1] Rydning DRJGJ, Reinsel J, Gantz J. The digitization of the world from edge to core, vol. 16. Framingham: International Data Corporation; 2018. p. 1–28.

[2] Kreis K, Neubauer S, Klora M, Lange A, Zeidler J. Status and perspectives of claims data analyses in germany—a systematic review. Health Pol (Amsterdam, Netherlands). 2016;120(2):213–26. 10.1016/j.healthpol.2016.01.007. 10.1016/j.healthpol.2016.01.007 26826756

[3] Blin P, Lassalle R, Thurin N, Bosco-Levy P, Droz-Perroteau C, Moore N. Snds, the french nationwide claims database: A powerful tool for pharmacoeconomy and pharmacoepidemiology. Value Health 21. 10.1016/j.jval.2018.09.221

[4] Novelli A, Frank-Teewag J, Bleek J, Guenster C, Schneider U, Marschall U, Schloessler K, Donner-Banzhoff N, Sundmacher L. Identifying and investigating ambulatory care sequences before invasive coronary angiography. Med Care 60. 10.1097/MLR.0000000000001738 10.1097/MLR.0000000000001738 PMC9257062 35700071

[5] Vanasse A, Courteau J, Courteau M, Benigeri M., Chiu YM, Dufour I, Couillard S., Larivee, P., Hudon, C.: Healthcare utilization after a first hospitalization for copd: a new approach of state sequence analysis based on the ‘6w’ multidimensional model of care trajectories. BMC Health Serv Res 2020;20(1). 10.1186/s12913-020-5030-0 10.1186/s12913-020-5030-0 PMC7059729 32143702

[6] Roux J, Grimaud O, Leray E. Care consumption of multiple sclerosis patients in france: an analysis of health insurance administrative databases using multichannel sequence analysis from 2007 to 2013. Mult Scler J 2018;23. 10.26226/morressier.59a3e8b4d462b8028d8942e9

[7] Lambert-Cote L, Bouhnik AD, Bendiane MK, Berenger C, Mondor M, Huiart L, Lauzier S (2020). Adherence trajectories of adjuvant endocrine therapy in the five years after its initiation among women with non-metastatic breast cancer: a cohort study using administrative databases. Breast Cancer Res Treat..

[8] Yan C, Chen Y, Li B, Liebovitz D, Malin B. Learning clinical workflows to identify subgroups of heart failure patients. AMIA Annu Symp Proc. 2017;2016:1248–57. PMC5333346 28269922

[9] Zhang Y, Padman R, Wasserman L, Patel N, Teredesai P, Xie Q (2015). On clinical pathway discovery from electronic health record data. IEEE ntell Syst..

[10] Lakshmanan GT, Rozsnyai S, Wang F. Investigating clinical care pathways correlated with outcomes. Lecture Notes in Computer Science (including subseries Lecture Notes in Artificial Intelligence and Lecture Notes in Bioinformatics). 2013;8094:323–38. 10.1007/978-3-642-40176-3_27

[11] Aspland E, Gartner D, Harper P. Clinical pathway modelling: a literature review. Health Syst (Basingstoke). 10.1080/20476965.2019.1652547 10.1080/20476965.2019.1652547 PMC7946019 33758656

[12] Williams R, Rojas E, Peek N, Johnson OA. Process mining in primary care: a literaturereview. Stud Health Technol Inform. 2018;247:376–80. 29677986

[13] Kusuma GP, Kurniati AP, Rojas E, McInerney CD, Gale CP, Johnson OA (2021). Process mining of disease trajectories: A literature review. Stud Health Technol Inf..

[14] Pinaire J, Azé J , Bringay S, Landais P. Patient healthcare trajectory. an essential monitoring tool: a systematic review. Health Inf Sci Syst. 10.1007/s13755-017-0020-2 10.1007/s13755-017-0020-2 PMC5390363 28413630

[15] Peters M, Godfrey C, Mcinerney P, Soares C, Khalil H, Parker D. Methodology for jbi scoping reviews, 1st ed. p. 1–24. Joanna Briggs Institute; 2015

[16] Tricco AC, Lillie E, Zarin W (2018). Prisma extension for scoping reviews (prisma-scr): Checklist and explanation. Ann Intern Med..

[17] Ouzzani M, Hammady H, Zbys F, Ahmed E (2016). Rayyan-a web and mobile app for systematic reviews. Syst Rev..

[18] Landis JR, Ritschard G. The measurement of observer agreement for categorical data. Biometrics 33(1):159–174. 10.2307/2529310 843571

[19] Alharbi A, Bulpitt A, Johnson OA, Klein GO, Karlsson D, Moen A, Ugon A (2018). Towards unsupervised detection of process models in healthcare. Stud Health Technol Inform..

[20] Baker K, Dunwoodie E, Jones RG, Newsham A, Johnson O, Price CP, Wolstenholme J, Leal J, McGinley P, Twelves C, Hall G (2017). Process mining routinely collected electronic health records to define real-life clinical pathways during chemotherapy. Int J Med Inform..

[21] Bobroske K, Larish C, Cattrell A, Bjarnadottir MV, Huan L (2020). The bird’s-eye view: A data-driven approach to understanding patient journeys from claims data. J Am Med Inform Assoc..

[22] Cerquitelli T, Chiusano S, Xiao X (2016). Exploiting clustering algorithms in a multiplelevel fashion: A comparative study in the medical care scenario. Expert Syst Appl..

[23] Charles-Nelson A, Lazzati A, Katsahian S (2020). Analysis of trajectories of care after bariatric surgery using data mining method and health administrative information systems. Obes Surg..

[24] Chen JF, Sun LL, Guo CH, Xie YM. A fusion framework to extract typical treatment patterns from electronic medical records. Artif Intell Med 2020;103. 10.1016/j.artmed.2019.101782 10.1016/j.artmed.2019.101782 32143789

[25] Chen JF, Sun LL, Guo CH, Wei W, Xie YM (2018). A data-driven framework of typical treatment process extraction and evaluation. J Biomed Inform..

[26] Cheng Q, Ren X, Yuan H, Geng J, Bian F. Medical insurance data mining using spam algorithm. 19th International Symposium on Knowledge and Systems Sciences, KSS 2018, vol 698, 2017. p. 100-108. 10.1007/978-981-10-3966-9_11

[27] Cherrie M, Curtis S, Baranyi G, McTaggart S, Cunningham N, Licence K, Dibben C, Bambra C, Pearce J. Use of sequence analysis for classifying individual antidepressant trajectories to monitor population mental health. BMC Psychiatr 2020;20(1). 10.1186/s12888-020-02952-y 10.1186/s12888-020-02952-y PMC7684902 33228576

[28] Chiudinelli L, Dagliati A, Tibollo V, Albasini S, Geifman N, Peek N, Holmes JH, Corsi F, Bellazzi R, Sacchi L (2020). Mining post-surgical care processes in breast cancer patients. Artif Intell Med..

[29] Concaro S, Sacchi L, Cerra C, Fratino P, Bellazzi R (2011). Mining health care administrative data with temporal association rules on hybrid events. Methods Inf Med..

[30] Dagliati A, Tibollo V, Cogni G, Chiovato L, Bellazzi R, Sacchi L. Careflow mining techniques to explore type 2 diabetes evolution. J Diabetes Sci Technol 201812(2):251–259. 10.1177/1932296818761751 10.1177/1932296818761751 PMC5851241 29493360

[31] Dauxais y, Guyet t, Gross-Amblard D, Happe A. Discriminant chronicles mining: Application to care pathways analytics. Artificial Intelligence in Medicine. Vienna: 2017. 10.1007/978-3-319-59758-4ff6ff.ffhal-01568929f.

[32] Egho E, Jay N, Raissi C, Ienco D, Poncelet P, Teisseire M, Napoli A (2014). A contribution to the discovery of multidimensional patterns in healthcare trajectories. J Intell Inf Syst..

[33] Esmaili N, Buchlak QD, Piccardi M, Kruger B, Girosi F. Multichannel mixture models for time-series analysis and classification of engagement with multiple health services: An application to psychology and physiotherapy utilization patterns after traffic accidents. Artif Intell Med 2021;111. 10.1016/j.artmed.2020.101997 10.1016/j.artmed.2020.101997 33461690

[34] Estiri H, Strasser ZH, Murphy SN. High-throughput phenotyping with temporal sequences. J Am Med Inform Assoc. 2020:1019970. 10.1093/jamia/ocaa288 10.1093/jamia/ocaa288 PMC7973443 33313899

[35] Han XY, Jiang F, Zhou HX, Needleman J, Guo MN, Chen Y, Liu YL, Tang YL (2020). Hospitalization pattern, inpatient service utilization and quality of care in patients with alcohol use disorder: A sequence analysis of discharge medical records. Alcohol Alcohol..

[36] Hilton R, Zheng YC, Fitzpatrick A, Serban N (2018). Uncovering longitudinal health care behaviors for millions of medicaid enrollees: A multistate comparison of pediatric asthma utilization. Med Decis Making..

[37] Honda Y, Kushima M, Yamazaki T, Araki K, Yokota H, Begoli E, Luo G, Wang F. Detection and visualization of variants in typical medical treatment sequences. Lecture Notes in Computer Science (including subseries Lecture Notes in Artificial Intelligence and Lecture Notes in Bioinformatics). 2017;10494:89–101. 10.1007/978-3-319-67186-4_8

[38] Hur C, Wi J, Kim Y. Facilitating the development of deep learning models with visual analytics for electronic health records. Int J Environ Res Public Health 2020;17(22). 10.3390/ijerph17228303 10.3390/ijerph17228303 PMC7697823 33182703

[39] Kempa-Liehr AW, Lin CYC, Britten R, Armstrong D, Wallace J, Mordaunt D, O’Sullivan M (2020). Healthcare pathway discovery and probabilistic machine learning. Int J Med Inform..

[40] Ku CC, Chen CC, Dixon S, Lin HH, Dodd PJ. Patient pathways of tuberculosis care-seeking and treatment: an individual-level analysis of national health insurance data in taiwan. BMJ Glob Health 2020;5(6). 10.1136/bmjgh-2019-002187 10.1136/bmjgh-2019-002187 PMC7307534 32565426

[41] Le H.H, Yamada T, Honda Y, Kayahara M, Kushima M, Araki K, Yokota H, Hartmann S, Kung J, Anderst-Kotsis G, Khalil I, Chakravarthy S, Tjoa AM. Analyzing sequence pattern variants in sequential pattern mining and its application to electronic medical record systems. Lect Notes Comput Sci. 2019;11707:393–408. 10.1007/978-3-030-27618-8_29.

[42] Le Meur N, Gao F, Bayat S. Mining care trajectories using health administrative information systems: the use of state sequence analysis to assess disparities in prenatal care consumption. BMC Health Serv Res 2015;15. 10.1186/s12913-015-0857-5 10.1186/s12913-015-0857-5 PMC4436876 25976089

[43] Li JQ, Tan XY, Xu X, Wang F (2019). Efficient mining template of predictive temporal clinical event patterns from patient electronic medical records. IEEE J Biomed Health Inf..

[44] Meng W, Ou W, Chandwani S, Chen X, Black W, Cai Z (2019). Temporal phenotyping by mining healthcare data to derive lines of therapy for cancer. J Biomed Inform..

[45] Najjar A, Reinharz D, Girouard C, Gagne C (2018). A two-step approach for mining patient treatment pathways in administrative healthcare databases. Artif Intell Med..

[46] Nuemi G, Afonso F, Roussot A, Billard L, Cottenet J, Combier E, Diday E, Quantin C (2013). Classification of hospital pathways in the management of cancer: Application to lung cancer in the region of burgundy. Cancer Epidemiol..

[47] Oh W, Kim E, Castro MR, Caraballo PJ, Kumar V, Steinbach MS, Simon GJ (2016). Type 2 diabetes mellitus trajectories and associated risks. Big Data..

[48] Ou-Young C, Chou SC, Juan YC, Wang HC. Mining sequential patterns of diseases contracted and medications prescribed before the development of stevens-johnson syndrome in taiwan. Appl Sci-Basel 2019;9(12). 10.3390/app9122434

[49] Perer A, Wang F, Hu JY (2015). Mining and exploring care pathways from electronic medical records with visual analytics. J Biomed Inform..

[50] Pokharel S, Zuccon G, Li Y. Representing EHRs with Temporal Tree and Sequential Pattern Mining for Similarity Computing. In: Yang, X., Wang, CD., Islam, M.S., Zhang, Z. (eds) Advanced Data Mining and Applications. ADMA 2020. Lecture Notes in Computer Science, vol 12447. Cham: Springer; 2020. 10.1007/978-3-030-65390-3_18.

[51] Rama K, Canhao H, Carvalho AM, Vinga S. Aliclu - temporal sequence alignment for clustering longitudinal clinical data. BMC Med Inform Dec Making 2019;19(1). 10.1186/s12911-019-1013-7 10.1186/s12911-019-1013-7 PMC6938005 31888660

[52] Rao A, Bottle A, Darzi A, Aylin P (2017). Sequence analysis of long-term readmissions among high-impact users of cerebrovascular patients. Stroke Res Treat..

[53] Rao A, Bottle A, Bicknell C, Darzi A, Aylin P. Common sequences of emergency readmissions among high-impact users following aaa repair. Surg Res Pract. 2018;1–11. 10.1155/2018/5468010 10.1155/2018/5468010 PMC6051028 30057940

[54] Rao G, Kirley K, Epner P, Zhang YY, Bauer V, Padman R, Zhou Y, Solomonides A (2018). Identifying, analyzing, and visualizing diagnostic paths for patients with nonspecific abdominal pain. Appl Clin Inf..

[55] Righolt CH, Zhang G, Mahmud SM (2020). Classification of drug use patterns. Pharmacol Res Perspect..

[56] Solomon DH, Xu C, Collins J, Kim SC, Losina E, Yau V, Johansson FD. The sequence of disease-modifying antirheumatic drugs: pathways to and predictors of tocilizumab monotherapy. Arthritis Res Ther 2020;23(1). 10.1186/s13075-020-02408-40 10.1186/s13075-020-02408-4 PMC7807904 33446261

[57] Sun W, Shen W, Li X, Cao F, Ni Y, Liu H. Mining information dependency in outpatient encounters for chronic disease care. 40th Medical Informatics in Europe Conference, MIE 2018, vol 192, 2013. p. 278–282. 23920560

[58] Vogt V, Scholz SM, Sundmacher L (2017). Applying sequence clustering techniques to explore practice-based ambulatory care pathways in insurance claims data. Eur J Public Health..

[59] Wang F, Lee N, Hu J, Sun J, Ebadollahi S, Laine AF (2013). A framework for mining signatures from event sequences and its applications in healthcare data. IEEE Trans Pattern Anal Mach Intel..

[60] Wright AP, Wright AT, McCoy AB, Sittig DF (2015). The use of sequential pattern mining to predict next prescribed medications. J Biomed Inform..

[61] Zhang Y, Padman R, Wasserman L (2014). On learning and visualizing practice-based clinical pathways for chronic kidney disease.

[62] Zhang YY, Padman R (2015). Innovations in chronic care delivery using data-driven clinical pathways. Am J Manage Care..

[63] Wilson J, Bock A. The benefit of using both claims data and electronic medical record data in health care analysis. Optum Insight. 2012;1:1–4.

[64] Cadarette S. An introduction to health care administrative data. Can J Hosp Pharm. 10.4212/cjhp.v68i3.1457 10.4212/cjhp.v68i3.1457 PMC4485511 26157185

[65] Centers for Medicare & Medicaid Services: Electronic Health Records. https://www.cms.gov/Medicare/E-Health/EHealthRecords. Accessed 03 Oct 2022

[66] Zhang Y, Padman R, Patel N (2015). Paving the cowpath: Learning and visualizing clinical pathways from electronic health record data. J Biomed Inform..

[67] World Health Organisation. International Statistical Classification of Diseases and Related Health Problems 10th Revision. https://icd.who.int/browse10/2019/en. Accessed 23 Jan 2023.

[68] Banda JM, Seneviratne M, Hernandez-Boussard T, Shah NH (2018). Advances in electronic phenotyping: From rule-based definitions to machine learning models. Ann Rev Biomed Data Sci..

[69] Hastie T, Tibshirani R, Friedman J. The Elements of Statistical Learning, 2nd ed. Springer

[70] Joe H, Ward Jr. Hierarchical grouping to optimize an objective function. J Am Stat Assoc. 1963;58(301):236–244. 10.1080/01621459.1963.10500845

[71] Nagin DS, Odgers CL. Group-based trajectory modeling in clinical research. Annu Rev Clin Psychol. 10.1146/annurev.clinpsy.121208.131413 10.1146/annurev.clinpsy.121208.131413 20192788

[72] Aggarwal C. An introduction to frequent pattern mining. In: Frequent Pattern Mining, p. 1–17. Springer. 10.1007/978-3-319-07821-2_1

[73] Ayres J, Flannick J, Gehrke J, Yiu T (2002). Sequential pattern mining using a bitmap representation..

[74] Zaki MJ (2001). Parallel sequence mining on shared-memory machines. J Parallel Distrib Comput..

[75] Dagliati A, Sacchi L, Zambelli A, Tibollo V, Pavesi L, Holmes JH, Bellazzi R (2017). Temporal electronic phenotyping by mining careflows of breast cancer patients. J Biomed Inform..

[76] Ching WK, Ng KP. Markov Chains: Models, Algorithms and Applications. New York: Springer; 2006. 10.1007/0-387-29337-X.

[77] Leemans SJJ, Fahland D, Aalst van der WMP. Exploring processes and deviations. In: Fournier F, Mendling J, editors. Business Process Management Workshops. Springer; 2015. p. 304–16

[78] Gabadinho A, Ritschard G, Studer M, Müller NS. Mining sequence data in R with the TraMineR package: A user’s guide. University of Geneva; 2010. http://mephisto.unige.ch/traminer.

