# Supplementary material for: Analytical methods for identifying sequences of utilization in health data: a scoping review
Source: BMC Med Res Methodol. 2023 Sep 27;23:212. doi: 10.1186/s12874-023-02019-y (PMC10523647; doi:10.1186/s12874-023-02019-y)
Supplement: Supplementary file 1 — Additional file 1. Search Strategy, Inclusion and Exclusion criteria, Cohen’s Kappa. Part I contains the defined inclusion and exclusion criteria against which the data sets are reviewed for eligibility. Part II contains the structure of the search strategy and the search strategies for each database. Part III contains data to calculate Cohen’s Kappa as measure for agreement in the screening process. [file 12874_2023_2019_MOESM1_ESM.pdf]

Additional File 1: Search strategy

Part I: Eligibility criteria (includes Inclusion and Exclusion criteria)

Part II: Full Search Strategies

Part III: Calculation of Cohen’s Kappa

Part I: Eligibility criteria (includes Inclusion and Exclusion criteria)

PICO-Scheme:

Population: Individuals whose health data are processed. No restriction in human population (patients, insured persons, other persons). No limitation on age, origin, morbidity and sex of population.

Intervention: Application or presentation of an implemented method that examines health data in terms of care sequences.

Comparison: No comparison.

Outcome: No specific outcome.

Inclusion:

Time: 01/01/2000 – 16/03/2021

Language: English

Databases: PubMed, Web Of Science, EconLit, Scopus

Setting: Health Care Sector.

Further criteria:
1. Database: Method must be applied to real world data, which include health care utilization data (e.g. Electronic Health Records, Medical Health Records, Claims data). Data must be individual patient-related data.
2. Method: The method used must be at least roughly described or, if it is a known method, named. Applied Method must consider the chronology of events. Considered events must include events of utilization.
3. Results: Sequences of care have to be presented in any way. Sketches and sections of the results are sufficient. A chronological sequence of events must be observed.

Exclusion:

Type of publication: No limitation.

Further criteria:
1. Data: Studies using Datasets of only medical values are excluded. Aggregated data in terms of time or populations (no individual patient-related data). Qualitative databases e.g. from questionnaires.
2. Method: Methods which are not explained or named at all. Methods of pattern recognition in terms of imaging techniques or text recognition. Purely technical articles without practical application.
3. Results: Results do not consider chronological order of events. All types of presentations are accepted.

Additional File 1: Search strategy

Part II: Full Search Strategies

Databases searched

Table 1: Overview of the search for matching articles in the databases.

Database	Date	Number of articles
Medline via PubMed	16/03/2021	840
Scopus	16/03/2021	2641
Web of Science	16/03/2021	856
EconLit	16/03/2021	6
Total (incl. Duplicates)		4343
**Total (excl. Duplicates)**		**2865**

General structure of search strategy

Table 2: Overview of the search strategy divided into the three subcategories: type of data, type of information and type of method.

1) type of data	2) type of information		3) type of method
	a)	b)	
administrative data /<br>administrative database /<br>administrative databases	patient /patients<br>care<br>clinical<br>ambulatory<br>treatment /<br>treatments<br>health<br>healthcare<br>stationary<br>hospital<br>inpatient<br>outpatient<br>utilization/<br>utilisation	trajectory/<br>trajectories<br>sequence /<br>sequences<br>sequential<br>pattern/<br>patterns<br>pathway/<br>pathways<br>journey/<br>journeys<br>path/ paths	algorithm mining /<br>mining algorithms<br>technique mining /<br>mining techniques<br>process mining / mining processes<br>pattern mining / mining patterns<br>itemset mining / mining itemsets<br>string mining / mining strings<br>sequence mining / mining sequences<br>event mining / mining events<br>rule mining / mining rules
pharmacy claims			data mining / mining data<br>data classification<br>data summarization<br>data summarisation
electronic health record / electronic health records			supervised learning<br>unsupervised learning<br>machine learning / machine-learning<br>deep learning
insurance claims			clustering /<br>cluster /clusters
claims data /<br>claims database /<br>claims databases			neural network /neural networks
patient data			decision tree / decision trees
real-world data /<br>real world data			patient pathway analysis /<br>patient-pathway analysis /<br>patient pathway analyses /<br>patient-pathway analyses

Additional File 1: Search strategy

routine data / database /databases		support vector machine support vector machines
patient record / records		sequence analysis / sequence analyses
insurance data / database /databases		
medical record / medical records		
administrative claims		

Specific search strategy per database

Table 3: Exact search strategy in the PubMed database.

**Database: PubMed**<br><br>**Search Date: 16.03.2021**		
	**Search Strategy: 1 AND 2a AND 2b AND 3 AND R**	**840**
**1**	(<br>("administrative data"[Title/Abstract]) OR ("administrative database"[Title/Abstract]) OR ("administrative databases"[Title/Abstract]) OR ("administrative claims"[Title/Abstract]) OR<br>("pharmacy claims"[Title/Abstract]) OR<br>("electronic health record"[Title/Abstract]) OR ("electronic health records"[Title/Abstract]) OR ("insurance claims"[Title/Abstract]) OR<br>("claims data"[Title/Abstract]) OR ("claims database"[Title/Abstract]) OR<br>("claims databases"[Title/Abstract]) OR<br>("patient data"[Title/Abstract]) OR<br>("real-world data"[Title/Abstract]) OR ("real world data"[Title/Abstract]) OR<br>("routine data"[Title/Abstract]) OR ("routine database"[Title/Abstract]) OR<br>("routine databases"[Title/Abstract]) OR<br>("patient record"[Title/Abstract]) OR ("patient records"[Title/Abstract]) OR<br>("medical record"[Title/Abstract]) OR ("medical records"[Title/Abstract]) OR<br>("insurance data"[Title/Abstract]) OR ("insurance database"[Title/Abstract]) OR ("insurance databases"[Title/Abstract])<br>)	
**2 a**	("healthcare"[Title/Abstract] OR "patient"[Title/Abstract] OR "patients"[Title/Abstract] OR "care"[Title/Abstract] OR "clinical"[Title/Abstract] OR "hospital"[Title/Abstract] OR "ambulatory"[Title/Abstract] OR "treatment"[Title/Abstract] OR "treatments"[Title/Abstract] OR "health"[Title/Abstract] OR "stationary"[Title/Abstract] OR "utilization"[Title/Abstract] OR "utilisation"[Title/Abstract] OR "inpatient"[Title/Abstract] OR "outpatient"[Title/Abstract]<br>)	
**2 b**	("sequences"[Title/Abstract] OR "sequence"[Title/Abstract] OR "patterns"[Title/Abstract] OR "pattern"[Title/Abstract] OR "pathways"[Title/Abstract] OR "pathway"[Title/Abstract] OR "journeys"[Title/Abstract] OR "journey"[Title/Abstract] OR "paths"[Title/Abstract] OR "path"[Title/Abstract] OR	

	"trajectory"[Title/Abstract] OR "trajectories"[Title/Abstract] OR "sequential"[Title/Abstract])	
**3**	("algorithm mining"[Title/Abstract] OR "mining algorithms"[Title/Abstract] OR "event mining"[Title/Abstract] OR "mining events"[Title/Abstract] OR "rule mining"[Title/Abstract] OR "mining rules"[Title/Abstract] OR "technique mining"[Title/Abstract] OR "mining techniques"[Title/Abstract] OR "process mining"[Title/Abstract] OR "mining processes"[Title/Abstract] OR "pattern mining"[Title/Abstract] OR "mining patterns"[Title/Abstract] OR "itemset mining"[Title/Abstract] OR "mining itemsets"[Title/Abstract] OR "string mining"[Title/Abstract] OR "mining strings"[Title/Abstract] OR "sequence mining "[Title/Abstract] OR "mining sequences"[Title/Abstract] OR "data mining"[Title/Abstract] OR "mining data"[Title/Abstract] OR "data classification"[Title/Abstract] OR "data summarization"[Title/Abstract] OR "data summarisation"[Title/Abstract] OR "supervised learning"[Title/Abstract] OR "unsupervised learning"[Title/Abstract] OR "machine learning"[Title/Abstract] OR "machine-learning"[Title/Abstract] OR "deep learning"[Title/Abstract] OR "clustering"[Title/Abstract] OR "neural network"[Title/Abstract] OR "neural networks"[Title/Abstract] OR "decision tree"[Title/Abstract] OR "decision trees"[Title/Abstract] OR "support vector machine"[Title/Abstract] OR "support vector machines"[Title/Abstract] OR "patient pathway analysis"[Title/Abstract] OR "patient pathway analyses"[Title/Abstract] OR "patient-pathway analysis"[Title/Abstract] OR "patient-pathway analyses"[Title/Abstract] OR "sequence analysis" [Title/Abstract] OR "sequence analyses" [Title/Abstract])	
**R**	(english[Filter] OR german[Filter]) AND (2000:2021[pdat])	

Table 4: Exact search strategy in the Scopus database.

	**Database: Scopus**<br><br>**Search Date: 16.03.2021**	
	**Complete Search Strategy: Search 1 AND Search 2**	**2,641**
	Search 1: 1 AND 2a AND 2b AND 3 AND R1	1,149
	Search 2: 1 AND 2a AND 2b AND 3 AND R2	1,492
**1**	TITLE-ABS-KEY ({administrative data} OR {administrative database} OR {administrative databases} OR {administrative claims} OR {pharmacy claims} OR {electronic health record} OR {electronic health records} OR {insurance claims} OR {claims data} OR {claims database} OR {claims databases} OR {patient data} OR {realworld data} OR {real world data} OR {routine data} OR {routine database} OR {routine databases} OR {patient record} OR {patient records} OR {medical record} OR {medical records} OR {insurance data} OR {insurance database} OR {insurance databases})	
**2 a**	TITLE-ABS-KEY ({healthcare} OR {patient} OR {patients} OR {care} OR {clinical} OR {hospital} OR {ambulatory} OR {treatment} OR {treatments} OR {health} OR {stationary} OR {utilization} OR {utilisation} OR {inpatient} OR {outpatient})	
**2 b**	TITLE-ABS-KEY ({sequences} OR {sequence} OR {patterns} OR {pattern} OR {pathways} OR {pathway} OR {journeys} OR {journey} OR {paths} OR {path} OR {trajectory} OR {trajectories} OR {sequential})	
**3**	TITLE-ABS-KEY ({algorithm mining} OR {mining algorithms} OR {event mining} OR {mining events} OR {rule mining} OR {mining rules} OR {technique mining} OR {mining techniques} OR {process mining} OR {mining processes} OR {pattern mining} OR {mining patterns} OR	

	{itemset mining} OR {mining itemsets} OR {string mining} OR {mining strings} OR {sequence mining } OR {mining sequences} OR {data mining} OR {mining data} OR {data classification} OR {data summarization} OR {data summarisation} OR {supervised learning} OR {unsupervised learning} OR {machine learning} OR {machine - learning} OR {deep learning} OR {clustering} OR {neural network} OR {neural networks} OR {decision tree} OR {decision trees} OR {support vector machine} OR {support vector machines} OR {patient pathway analysis} OR {patient pathway analyses} OR {patient - pathway analysis} OR {patient - pathway analyses} OR {sequence analysis} OR {sequence analyses})	
**R1**	LANGUAGE (english OR german)<br>AND PUBYEAR > 1999 AND PUBYEAR < 2016	
**R2**	AND LANGUAGE (english OR german)<br>AND PUBYEAR > 2015	

Table 5: Exact search strategy in the EconLit database.

	**Database: EconLit**<br><br>**Search Date: 16.03.2021**	
	**Search Strategy: 1 AND 2a AND 2b AND 3 AND R**	**6**
**1**	AB (<br>("administrative data" OR "administrative database" OR "administrative databases" OR "administrative claims" OR "pharmacy claims" OR "electronic health record" OR “electronic health records" OR "insurance claims" OR "claims data" OR "claims database" OR "claims databases" OR "patient data" OR "real-world data" OR “real world data" OR "routine data" OR "routine database" OR "routine databases" OR "patient record" OR "patient records" OR "medical record" OR "medical records" OR "insurance data" OR "insurance database" OR "insurance databases")<br>)	
**2 a**	AB (<br>("healthcare" OR "patient" OR "patients" OR "care" OR "clinical" OR "hospital" OR "ambulatory" OR "treatment" OR "treatments" OR "health" OR “stationary" OR "utilization" OR "utilisation" OR "inpatient" OR "outpatient")<br>)	
**2 b**	AB (<br>("sequences" OR "sequence" OR "patterns" OR "pattern" OR "pathways" OR "pathway" OR "journeys" OR "journey" OR "paths" OR "path" OR "trajectory" OR "trajectories" OR "sequential")<br>)	
**3**	AB (<br>("algorithm mining" OR "mining algorithms" OR<br>"event mining" OR "mining events" OR<br>"rule mining" OR "mining rules" OR<br>"technique mining" OR "mining techniques" OR<br>"process mining" OR "mining processes" OR<br>"pattern mining" OR "mining patterns" OR<br>"itemset mining" OR "mining itemsets" OR<br>"string mining" OR "mining strings" OR<br>"sequence mining" OR "mining sequences" OR<br>"data mining" OR "mining data" OR<br>"data classification" OR	

	"data summarization" OR "data summarisation" OR "supervised learning" OR "unsupervised learning" OR "machine learning" OR "machine-learning" OR "deep learning" OR "clustering" OR "neural network" OR "neural networks" OR "decision tree" OR "decision trees" OR "support vector machine" OR "support vector machines" OR "patient pathway analysis" OR "patient pathway analyses" OR "patient-pathway analysis" OR "patient-pathway analyses" OR "sequence analysis" OR "sequence analyses"))	
**R**	LA ((english OR german)) **Limiters** - Published Date: 20000101-20210331 **Search modes** - Find any of my search terms	

Table 6: Exact search strategy in the Web of Science database.

	**Database: Web of Science** **Search Date: 16.03.2021**	
	**Search Strategy: 1 AND 2a AND 2b AND 3 AND R**	**856**
**1**	TOPIC: (("administrative data" OR "administrative database" OR "administrative databases" OR "administrative claims" OR "pharmacy claims" OR "electronic health record" OR "electronic health records" OR "insurance claims" OR "claims data" OR "claims database" OR "claims databases" OR "patient data" OR "real-world data" OR “real world data" OR "routine data" OR "routine database" OR "routine databases" OR "patient record" OR "patient records" OR "medical record" OR "medical records" OR "insurance data" OR "insurance database" OR "insurance databases"))	
**2 a**	TOPIC: (("healthcare" OR "patient" OR "patients" OR "care" OR "clinical" OR "hospital" OR "ambulatory" OR "treatment" OR "treatments" OR "health" OR "stationary" OR "utilization" OR "utilisation" OR "inpatient" OR "outpatient"))	
**2 b**	TOPIC: (("sequences" OR "sequence" OR "patterns" OR "pattern" OR "pathways" OR "pathway" OR "journeys" OR "journey" OR "paths" OR "path" OR "trajectory" OR "trajectories" OR "sequential"))	
**3**	TOPIC: (("algorithm mining" OR "mining algorithms" OR "event mining" OR "mining events" OR "rule mining" OR "mining rules" OR "technique mining" OR "mining techniques" OR "process mining" OR "mining processes" OR "pattern mining" OR "mining patterns" OR	

	"itemset mining" OR "mining itemsets" OR<br>"string mining" OR "mining strings" OR<br>"sequence mining " OR "mining sequences" OR<br>"data mining" OR "mining data" OR<br>"data classification" OR<br>"data summarization" OR "data summarisation" OR<br>"supervised learning" OR "unsupervised learning" OR<br>"machine learning" OR "machine-learning" OR<br>"deep learning" OR "clustering" OR<br>"neural network" OR "neural networks" OR<br>"decision tree" OR "decision trees" OR<br>"support vector machine" OR "support vector machines" OR<br>"patient pathway analysis" OR "patient pathway analyses" OR<br>"patient-pathway analysis" OR "patient-pathway analyses" OR<br>"sequence analysis" OR "sequence analyses")<br>)	
**R**	LANGUAGE: (English OR German)<br>Timespan: 2000-2021. Indexes: SCI-EXPANDED, SSCI	

PART III: Calculation of Cohen's Kappa

Phase 1:
Decisions of the two reviewers in stage one: Screening of abtracts and titles.

Table 7: Overview of the inclusion and exclusion decisions of the two reviewers (A.F. and A.N.) in the first decision phase.

**reviewers**		**A.N.**		
	**decision**	**IN**	**OUT**	
**A.F.**	**IN**	98	17	115
	**OUT**	23	2727	2750
		121	2744	**2865**

Kappa = 0.8232 which corresponds to 82 %.

Phase 2:
Decisions of the two reviewers in stage two: Screening of the full texts.

Table 8: Overview of the inclusion and exclusion decisions of the two reviewers (A.F. and A.N.) in the second decision phase.

**reviewers**		**A.N.**		
	**decision**	**IN**	**OUT**	
**A.F.**	**IN**	48	7	55
	**OUT**	3	62	65
		51	69	**120**

Kappa = 0.8312 which corresponds to 83 %.
