# Supplementary material for: Analytical methods for identifying sequences of utilization in health data: a scoping review
Source: BMC Med Res Methodol. 2023 Sep 27;23:212. doi: 10.1186/s12874-023-02019-y (PMC10523647; doi:10.1186/s12874-023-02019-y)
Supplement: Supplementary file 2 — Additional file 2. Data extraction. Part I includes extracted general information, Data Source, Setting and Population of the included articles, Part II includes the used events for constructing the sequences per included study and Part III includes information about the applied methodology, the Software, presentation of results and the stated aim of the study. [file 12874_2023_2019_MOESM2_ESM.pdf]

Additional File 2: Data Extraction of the included studies

Table 1: Extracted Information on Data Source, Setting and Population in the included studies.

Study	Type of data	Classification	Data horizon	Date of first data collection	Population size	origin of data / population	characteristic of population	corresponding ICD-10 chapter
Alharbi et al., 2018	US hospital database (MIMIC-III)	MIMIC-III	not named	not named	356	USA	altered mental status	18
Baker et al., 2017	EHR - cancer specific	EHR	about 10 years	2004	955	UK	cancer (diagnosis of metastatic breast cancer and received adjuvant epirubicin and cyclosphosphamide chemotherapy or (ii) colorectal cancer and received palliative oxaliplatin and infusional 5-fluorouracil chemotherapy, and (iii) were first diagnosed with cancer between January 2004 and February 2013)	2

Additional File 2: Data Extraction of the included studies

Study	Type of data	Classification	Data horizon	Date of first data collection	Population size	origin of data / population	characteristic of population	corresponding ICD-10 chapter
Boborske et al., 2020	claims data	claims data	79 months	2012	9,981	USA	back pain (patients who follow conservative back pain guidelines)	13
Cerquitelli et al., 2016	collection of patient records from Hospital	EMR	1 year	2014	6,38	Italy	diabetes (overt diabetes)	4
Charles-Nelson et al., 2020	administrative claims database (PMSI)	claims data	1 year	2013	196,323	France	obesity with bariatric surgery (certain surgeries named), diagnosis of obesity	4
Chen et al., 2020	EMR data from three chinese hospitals	EMR	14 days	not named	8,151	China	cerebral infarction	9
Chen et al., 2018	EMR data from three chinese hospitals	EMR	14 days	not named	8,287	China	cerebral infarction	9

Additional File 2: Data Extraction of the included studies

Study	Type of data	Classification	Data horizon	Date of first data collection	Population size	origin of data / population	characteristic of population	corresponding ICD-10 chapter
Cheng, Ren, 2017	health care data ; Medicare data	EMR	not named		8,287	not named	pregnancy	15
Cherrie et al., 2020	National Health Service (NHS) prescription data	claims data	6 months	2009-2014	151,418	Scotland	mental illness	5
Chiudinelle et al., 2020	Hospital Information Systems (HIS) and registry of clinical and molecular data: electronic health records	EHR	11 years	2007	3,564	Italy	cancer (breast cancer)	2

Additional File 2: Data Extraction of the included studies

Study	Type of data	Classification	Data horizon	Date of first data collection	Population size	origin of data / population	characteristic of population	corresponding ICD-10 chapter
Concaro et al., 2011	administrative database to the regional health care agency (ASL) of Pavia	administrative data	2 years	2005	2,513	Italy	diabetes	4
Dagliati et al., 2018	administrative data from Pavia Local Health Care Agency (ATS)	administrative data	10 years	after 2014	424	Italy	Diabetes (type 2)	4
Dauxais et al., 2017	SNIIRAM - more detailed about care events	SNIIRAM	3 years	2009	8,379	France	epilepsy	6
Egho et al., 2014	PMSI including administrative data and EMR	PMSI	not named	not named	828	France	cancer (lung cancer)	2

Additional File 2: Data Extraction of the included studies

Study	Type of data	Classification	Data horizon	Date of first data collection	Population size	origin of data / population	characteristic of population	corresponding ICD-10 chapter
Esmaili et al., 2021	accident data + utilization data of psychology and physiotherapy<br><br>Australien Transport Accident Commission	else	106 months		582	Australia	motor vehical accident	20
Estiri et al., 2020	EHR + medication records (Mass General Brigham Biobank)	EHR + medication records	5 years	2009	351	USA	several diseases (Alzheimer's disease, chronic obstructive pulmonary disease (COPD), congestive heart failure, coronary artery disease (CAD), stroke, rheumatoid	18, 13, 10, 9, more

Additional File 2: Data Extraction of the included studies

Study	Type of data	Classification	Data horizon	Date of first data collection	Population size	origin of data / population	characteristic of population	corresponding ICD-10 chapter
							arthritis, T1DM, T2DM, ulcerative colitis, and atrial fibrillation.)	
Han et al., 2020	discharge medical record database, Beijing	EMR	3 years		831	China	alcohol use disorder	5
Hilton et al., 2018	Medicaid Analytical Extract (MAX) medical claims data; individual-level claims data	(medical) claims data	several years	2012	1,500,000	USA	persistent asthma (children with persistant asthma; children ages 4–18 with an asthma-related diagnosis age: 4 - 18 years; pediatric asthma)	10

Additional File 2: Data Extraction of the included studies

Study	Type of data	Classification	Data horizon	Date of first data collection	Population size	origin of data / population	characteristic of population	corresponding ICD-10 chapter
Honda et al., 2017	EMR - medical treatment data - University of Miyazaji Hospital	EMR	24 years	1991	288	Japan	Resection of a Bladder tumor (TUR-Bt) und Endoscopic Submucosal Dissection (ESD)	2
Hur et al., 2020	Electronic health record - MIMIC-III database	MIMIC III	not named	not named	11,811	USA	heart disease	9
Kempa-Liehr et al., 2020	electronic patient records (from Radiology Information System (RIS) and the patient administration system iPM)	EMR + administrative data	2 years	2015	448	New Zealand	surgery	else

Additional File 2: Data Extraction of the included studies

Study	Type of data	Classification	Data horizon	Date of first data collection	Population size	origin of data / population	characteristic of population	corresponding ICD-10 chapter
Ku et al., 2020	National Health Insurance Research Database (comprises healthcare claims) routine data	claims data	10 years	2000	6,258	Taiwan	tuberculosis	1
Lakshmanan et al., 2013	EMR of an US-based health care provider	EMR	several years	not named	2,094	USA	heart disease (congestive heart failure (CHF))	9
Lambert-Côté et al., 2020	French national study VICAN2 (VIe deux ans après un diagnostic de CANcer): 1. SNIIRAM; 2. telephone interviews	SNIIRAM	5 years	2010	674	France	cancer (breast cancer,hormonesensitive)	2

Additional File 2: Data Extraction of the included studies

Study	Type of data	Classification	Data horizon	Date of first data collection	Population size	origin of data / population	characteristic of population	corresponding ICD-10 chapter
Le Meur et al., 2015	SNIIRAM	SNIIRAM	9 months	2008	2,518	France	pregnancy (who gave birth without complications)	15
Le et al., 2019	electronic medical records	EMR	24 years	1991	394	Japan	Transurethral Resection of a Bladder tumor (TUR-Bt)	2
Li et al., 2019	electronic medical records	EMR	4 years	--	4,977	not named	heart failure	9
Meng et al., 2019	IBM® MarketScan-commercial - insurance claims dataset	claims data - commercial	1 year + remaining time;<br>up to 8 years	2011	23,830	USA	cancer (metastatic nonsmall cell lung cancer and metastatic melanoma)	2
Najjar et al., 2018	administrative data (RAMQ, MSSS)	administrative data	9 years	2000	180,027	Canada	heart failure (heart failure in elderly people (older 65 years))	9
Nuemi et al., 2013	national PMSI databases	PMSI	1 year	2004	495	France	cancer (primary lung cancer who had undergone surgery for the management of non-metastatic	2

Additional File 2: Data Extraction of the included studies

Study	Type of data	Classification	Data horizon	Date of first data collection	Population size	origin of data / population	characteristic of population	correspondin g ICD-10 chapter
							malignant tumours)	
Oh et al., 2016	electronic health record from Mayo Clinic primary care patients residing in Olmsted County, MN	EHR	15 years	1999	43,509	USA	diabetes	4
Ou-Yang et al., 2019	Taiwan Health insurance database; NHIRD data	claims data	1 year	2008	545	Taiwan	SJS	12

Additional File 2: Data Extraction of the included studies

**Study**	**Type of data**	**Classification**	**Data horizon**	**Date of first data collection**	**Population size**	**origin of data / population**	**characteristic of population**	**correspondin g ICD-10 chapter**
Perer et al., 2015	EMR - source not clear - Dr Sorrentino (Chief Medical Officer of Providence Medical Foundations)	EMR	455 days	not named	1,386	not named	diabetes + hyperlipidemia diagnosis	4
Pokharel et al., 2020	MIMIC III dataset, including ICU data	MIMIC III	not named	not named	5,274	USA	sequential organ failure	1
Rama et al., 2019	EMR; Portuguese nationwide database developed by the Portuguese Society of Rheumatology	EMR	not named	not named	426	Portugal	rheumatoid arthritis	13

Additional File 2: Data Extraction of the included studies

Study	Type of data	Classification	Data horizon	Date of first data collection	Population size	origin of data / population	characteristic of population	corresponding ICD-10 chapter
Rao A. et al., 2018	administrative data: Hospital Episode Statistics data (HES) (include information on all the inpatient hospital admissions - public hospitals)	administrative data	5 years	2006	16,973	England	abdominal aortic aneurysm (after abdominal aortic aneurysm (AAA) repair)	9
Rao A. et al., 2017	national administrative data (Hospital Episode Statistics (HES))	administrative data	at least 4 years	2010	60,225	England	Cardiovascular diseases (cerebrovascular patients)	9
Rao G. et al., 2018	EHR: Enterprise Data Warehouse (EDW),	EHR	at least 12 months	2011	501	USA	abdominal pain	18

Additional File 2: Data Extraction of the included studies

Study	Type of data	Classification	Data horizon	Date of first data collection	Population size	origin of data / population	characteristic of population	correspondin g ICD-10 chapter
Righolt et al., 2020	Manitoba Health maintains several centralized, administrative electronic databases	administrative data	follow up 1,2,5,10 years	1995	27,786	Canada	Diabetes (type 2, usind metformin)	4
Roux et al., 2018	PMSI	PMSI	7 years	2007	1,000	France	multiple sclerosis	6
Solomon et al., 2020	Corrona, a large real-world Rheumatoid Arthritis registry	EMR	5 years	2002	7,300	USA	rheumatoid arthritis	13
Sun et al., 2013	outpatient encounter data	Not clear	1 year	2011	11,946	China	Diabetes (type 2)	4
Vanasse et al., 2020	health insurance board	Not clear	1 year	2013	2,581	Canada	COPD (Chronic Obstructive Pulmonary Disease)	10

Additional File 2: Data Extraction of the included studies

Study	Type of data	Classification	Data horizon	Date of first data collection	Population size	origin of data / population	characteristic of population	correspondin g ICD-10 chapter
Vogt et al., 2017	routine data; ambulatory sector	claims data	2 years	2009	982	Germany	heart disease (heart failure)	9
Wang et al., 2013	EHR specification of source	EHR	1 year	not named	21,384	USA	diabetes	4
Wright et al., 2005	claims data	claims data	3 years	2008	161,497	USA	diabetes	4
Yan et al., 2016	Data from EMR system	EMR	4 months	2010	785	USA	heart disease (heart failure)	9
Zhang and Padman, 2015	EHR data from a community nephrology practice in western Pennsylvania	EHR	4 years	2009	664	not named	CKD (chronic kidney disease)	14
Zhang, Padman, Wasserman, Patel, Teredesai and Xie, 2015	EHR	EHR	not named	2009	1,624	USA	CKD (chronic kidney disease)	14

Study	Type of data	Classification	Data horizon	Date of first data collection	Population size	origin of data / population	characteristic of population	corresponding ICD-10 chapter
Zhang, Padman and Patel, 2015	EHR - visit data	EHR	54 months	2009	1,576	USA	CKD (chronic kidney disease)	14
Zhang et al., 2014	EHR: office visit data from nephrology practice in Western Pennsylvania	EHR	4 years	2009	2,511	USA	CKD (chronic kidney disease)	14

Table 2: Extracted Information on used events for constructing the sequences in the included studies

Study	Care events	Prescriptions	Medi-cation	Medi-cation use	Proce-dures	visits	attended specialist	Hospit-alization	Diag-nosis	vital signs	health outcome	else
Alharbi et al., 2018	extraction of event logs											x
Baker et al., 2017	diagnosis								x			

Additional File 2: Data Extraction of the included studies

Study	Care events	Prescriptions	Medi-cation	Medi-cation use	Proce-dures	visits	attended specialist	Hospit-alization	Diag-nosis	vital signs	health outcome	else
Boborske et al., 2020	prescriptions, lab tests, visits	x			x	x						
Cerquitelli et al., 2016	prescriptions, examinations	x			x							
Charles-Nelson et al., 2020	diagnosis, procedures (therapeutical, medical)				x				x			
Chen et al., 2020	treatments, doctor order, lab results	x			x					x		
Chen et al., 2018	medication prescriptions, health outcome	x	x								x	
Cheng and Ren., 2017	medication, type of treating staff		x				x					
Cherrie et al., 2020	prescription data	x										
Chiudinelle et al., 2020	therapy, type of surgery, cancer specific data	x			x							
Concaro et al., 2011	DRG, diagnosis and procedures in hospital,		x		x				x			x

Additional File 2: Data Extraction of the included studies

Study	Care events	Prescriptions	Medi-cation	Medi-cation use	Proce-dures	visits	attended specialist	Hospit-alization	Diag-nosis	vital signs	health outcome	else
	ambulatory, drugs											
Dagliati et al., 2018	drug purchases, procedures (ambulatory), hospitalizations, SPU visits (short procedure unit)			x	x			x				
Dauxais et al., 2017	drugs, hospitalisations		x					x				
Egho et al., 2014	procedures, diagnosis (in hospital)				x				x			
Esmaili et al., 2021	treatments (psychology and physiotherapy)				x							
Estiri et al., 2020	diagnosis, medication,		x						x			
Han et al., 2020	medical records											x
Hilton et al., 2018	(selected) medication, (hospital, physician) visits		x			x						

Additional File 2: Data Extraction of the included studies

Study	Care events	Prescriptions	Medi-cation	Medi-cation use	Proce-dures	visits	attended specialist	Hospit-alization	Diag-nosis	vital signs	health outcome	else
Honda et al., 2017	treatments, diagnosis, medication		x		x				x			
Hur et al., 2020	diagnosis, comorbidities, lab tests, prescriptions, procedures	x			x				x			
Kempa-Liehr et al., 2020	patient records (hospital)											x
Ku et al., 2020	treatments, visits, healthcare records				x	x						x
Lakshmanan et al., 2013	diagnosis, medication, lab tests, vital signs		x		x				x	x		
Lambert-Côté et al., 2020	medication use, comorbidity			x								
Le Meur et al., 2015	visits					x						
Le et al., 2019	treatments, prescriptions	x			x							
Li et al., 2019	diagnosis, medication,		x		x				x			

Additional File 2: Data Extraction of the included studies

Study	Care events	Prescriptions	Medi-cation	Medi-cation use	Proce-dures	visits	attended specialist	Hospit-alization	Diag-nosis	vital signs	health outcome	else
	procedures, lab tests											
Meng et al., 2019	medications and specific procedures		x		x							
Najjar et al., 2018	(medical + hospital) visits, consultations					x						
Nuemi et al., 2013	hospital visits, procedures, specific disease information				x	x				x		
Oh et al., 2016	diagnosis, lab measurements, vitals								x	x		
Ou-Yang et al., 2019	(inpatient, ambulant) visits, prescriptions, diagnosis,	x				x			x			
Perer et al., 2015	medication, diagnosis, labs, vital signs		x		x				x	x		

Additional File 2: Data Extraction of the included studies

Study	Care events	Prescriptions	Medi-cation	Medi-cation use	Proce-dures	visits	attended specialist	Hospit-alization	Diag-nosis	vital signs	health outcome	else
Pokharel et al., 2020	ICU admissions, disease specific variables							x		x		
Rama et al., 2019	medication		x									
Rao A. et al., 2018	hospital visits, procedures and diagnosis during the hospital stay				x	x			x			
Rao A. et al., 2017	diagnosis, hospital visits,					x			x			
Rao G. et al., 2018	diagnosis, examinations				x				x			
Righolt et al., 2020	prescriptions, (physicians) visits	x				x						
Roux et al., 2018	(physicians, hospital) visits, medication, specific treatments and therapies		x		x	x						
Solomon et al., 2020	medication		x									

Additional File 2: Data Extraction of the included studies

**Study**	**Care events**	**Prescriptions**	**Medication**	**Medication use**	**Procedures**	**visits**	**attended specialist**	**Hospitalization**	**Diagnosis**	**vital signs**	**health outcome**	**else**
Sun et al., 2013	diagnosis, medication, lab tests (+ imaging reports)		x		x				x			
Vanasse et al., 2020	type of treating institution, type of care provider, consultation,					x	x					
Vogt et al., 2017	prescriptions, examinations, type of physician	x			x		x					
Wang et al., 2013	procedures, lab results, physician visits (primary)				x	x				x		
Wright et al., 2005	drug class		x									
Yan et al., 2016	diagnosis, procedures, visits				x	x			x			
Zhang and Padman, 2015	medications, diagnosis, biochemical measurements		x						x	x		
Zhang, Padman,	procedures, diagnosis,		x		x	x			x			

Additional File 2: Data Extraction of the included studies

Study	Care events	Prescriptions	Medi-cation	Medi-cation use	Proce-dures	visits	attended specialist	Hospit-alization	Diag-nosis	vital signs	health outcome	else
Wasserman, Patel, Teredesai and Xie, 2015	medication, visit purpose											
Zhang, Padman and Patel, 2015	procedures, medications, diagnosis, visits purpose		x		x	x			x			
Zhang et al., 2014	procedures, diagnosis, visits in hospital				x	x			x			

For presentation in Scoping Review the data was combined according to the following list:

Table 3: Aggregated information on the considered events.

Types of events for analysis		N (%)
	prescriptions	11 (22 %)
	medications, medication use	21 (41%)
	procedures	28 (55%)
	visits, hospitalization, attended specialist	23 (45%)
	diagnosis	21 (41%)
	individual health data: health outcome, results of lab/tests	9 (18%)

	Else: not specified (medical records, patient records) / others (DRGs)	5 (10%)

Table 4: Extracted data from the included studies. Information on applied methods and stated aim. Classifications of methods (MM: Markov Model, PM: Pattern Mining, Clust: Clustering) and Stated Aims (Proposal, Trajectory, Pattern, Prediction) given in the Scoping Review are added for clarity.

Study	Applied method	Method - class	Prediction	Stated aim	Aim - class	Presentation of results	Program use
Alharbi et al., 2018	Hidden Markov Model; parameter estimation with seqHMMR in R, chose best model on BIC; Viterbi-Algorithm to extract most probable sequences; use BIC to find best final log likelihood, fitting training and test data	MM		Exploration of hidden sub-processes	Pattern	pattern as weighted graph, flowchart	R, DISCO process mining
Baker et al., 2017	simplify pathways on experts opinions; Markov Model for transition	MM	no	Establishing of a reproducible process-mining methods; Define and quantify patient pathways during the treatment	Proposal, Trajectory	table including trajectories with weight; first steps of utilization	SQL
Boborske et al., 2020	ensemble clustering using different partitioning cluster algorithms (eg. K-means, kmeans++), Levenshtein edit distance for similarity; use kmeans++ to choose cluster centers.	Clust.	no	Development of method to extract patient care trajectory	Proposal, Trajectory	vizualized as sankey diagram	not named

Additional File 2: Data Extraction of the included studies

Study	Applied method	Method - class	Prediction	Stated aim	Aim - class	Presentation of results	Program use
Cerquitelli et al., 2016	Multi-Level-Clustering. similarity: Cosine distance measure for similarity. Clustering: Bisecting K-medoids, bisecting kmeans, refined kmeans and refined kmedoids; Multiple Level DBSCAN. Evaluation: Sum of squared error, Silhouette, overall similarity, Rand Index; MLC framework in Java, max. sequential pattern mining	Clust.		Proposal of two-phase data mining method.<br><br>Iteratively identification of groups of objects with common properties.	Proposal, Phenotyping	table including pattern with weights (support)	Java, RapidMiner
Charles-Nelson et al., 2020	Formal Concept Analysis includes a clustering of objects	Clust.	no	Define trajectory of care; Quantification of complications in treatment	Trajectory	directed graph, trajectory, weighted	Coron System, R
Chen et al., 2020	constructuin of DOSS; multiview Network Fusion; Markov Model; consideration of duration; clustering (spectral, k-means, laplacian)	MM	no	Discovery of treatment patterns to find right treatment	Proposal, Pattern	pattern as weighted graph, sequence on timeline	Matlab, R
Chen et al., 2018	construction of DOSS; Markov Models; clustering on DOS	MM	using treatment efficacy (outcome, economic, length of stay)	Extraction of treatment processes; For clinicians to make better decisions	Trajectory	trajectory with weights, directed	Matlab, R + Microsoft Visio
Cheng and Ren, 2017	Pattern Mining using Sequential Pattern Mining with bitmap representation (SPAM)	PM	no	Discovery of patients medical behavior	Pattern	table including pattern	not named
Cherrie et al., 2020	clustering on pattern; similarity: Optimal Matching for sequences; clustering: hierarchical using Wards method; Use of TraMineR in R	Clust.	determine the association between each antidepressant	Classification of individual treatment trajectories;	Trajectory, Phenotyping	trajectory on timeline	R, lme4

Additional File 2: Data Extraction of the included studies

Study	Applied method	Method - class	Prediction	Stated aim	Aim - class	Presentation of results	Program use
			trajectory (i.e. dependent variables) and each of the auxiliary measures of mental illness using multilevel logistic regression	Identification of groups of individuals with similar patterns of treatment; Characterizing population most at risk of increasing mental ill health.			
Chiudinelli et al., 2020	Topic Modelling to reduce complexity; careflow Mining (SPM); grouping by experts	PM	survival probability : Kaplan-Meier methods	Identify frequent patterns of care; Discovery temporal phenotypes.	Pattern, Phenotyping	trajectory as graph	R, Pentaho Kettle
Concaro et al., 2011	Association Rule Mining	Other		Proposal of method to extract temporal association rules on sequences using hybrid events	Proposal, Pattern	table with patterns and weights	not named
Dagliati et al., 2018	Pattern Mining using Careflow Mining based on Frequent Pattern Mining; clustering of careflows	PM		Identify frequent patterns of care	Pattern	trajectory as graph, directed	not named
Dauxais et al., 2017	Temporal Pattern Mining using discriminant chronicles (DCM); frequent itemset mining and rule Mining	PM	Identification of possible associations between hospitalizations for seizure and anti-epileptic drug switches	Proposal of new discriminant pattern mining algorithm; Discovering of discriminant chronicle patterns.	Proposal, Pattern, Prediction	patterns as graph, weighted; trajectories in table	not named

Additional File 2: Data Extraction of the included studies

Study	Applied method	Method - class	Prediction	Stated aim	Aim - class	Presentation of results	Program use
			in care pathway of epileptic patients. The DCM algorithm is used to extract patterns of drugs deliveries that discriminate occurrences of recent seizure.				
Egho et al., 2014	Pattern Mining - Mining Multidimensional Itemset Sequential Pattern	PM	nn	Identification of pattern from patient trajectories	Pattern	pattern with weights in table	Java
Esmaili et al., 2021	Markov Models for time series clustering; improve clustering using ML	MM	no	Identification of utilization patterns; Classification of patient groups associated with utilization patterns	Pattern, Phenotyping	trajectory with weights as graph	R, Python
Estiri et al., 2020	transitive Sequential Pattern Mining with minimize sparsity and maximize relevance algorithm (MSMR) to reduce complexity. R code online in github	PM	Yes. Disease prediction and classification; Bootstrap CV and log Regression	Proposal of phenotyping method; Analysis of temporal Representation mined through SPM improves phenotyping	Proposal, Phenotyping	patterns as graph, descriptive	R
Han et al., 2020	construction of pathways; discrete sequential-state analyis; clustering of trajectories. Similarity: Optimal Matching. Clustering: hierachical,	Clust.	Kruskal–Wallis tests and Mann–Whitney	Discovering of temporal hospitalization patterns	Trajectory	trajectory as graph	R

Additional File 2: Data Extraction of the included studies

Study	Applied method	Method - class	Prediction	Stated aim	Aim - class	Presentation of results	Program use
	Wards method. State Sequence Analysis with TraMineR in R.		U tests were used on continuous variables, and Chi-square tests were applied to the group comparisons among categorical variables. Pairwise comparisons were performed if applicable, with the Bonferroni correction. Our findings show the associations between the hospitalization patterns and healthcare service utilization, outcome quality and provider variations				

Additional File 2: Data Extraction of the included studies

**Study**	**Applied method**	**Method - class**	**Prediction**	**Stated aim**	**Aim - class**	**Presentation of results**	**Program use**
Hilton et al., 2018	Markov Models for transition; clustering of resulted sequences	MM		Proposal of framework for analyzing and vizualizing utilization	Proposal, Trajectory	trajectory with weights as graph	not named
Honda et al., 2017	Pattern Mining unsing T-PrefixSpan including the consideration of time intervalls between treatments; grouping of pathways on number of tratments per relative treatment day	PM	no	Proposal of method to detect variants in frequent patterns; Discovery of variants of frequent patterns of care; considering time information	Proposal, Pattern	trajectory as graph; patterns in table with weights	tool D3.js
Hur et al., 2020	Sequential Pattern Mining using CloFast to select groups of Patients	PM	LSTM attention model, a deep learning method, learning long-term dependencies; CloFAST interpretable for prediction of diagnosis of heart failure and probability of unplanned cardiac surgery; applying Cross validation	Proposal of method to increase efficiency iteratively data methods entailing sequence mining; Prediction model of unplanned surgeries	Proposal, Trajectory, Prediction	trajectories as graph - sankey; pattern in table with weights	SQL, Python, Keras + ReactJS

Additional File 2: Data Extraction of the included studies

**Study**	**Applied method**	**Method - class**	**Prediction**	**Stated aim**	**Aim - class**	**Presentation of results**	**Program use**
Kempa-Liehr et al., 2020	grouping of events to reduce complexity; Use ProM to discover and review healthcare pathways	Other	Machine learning methodologies based on probabilistic programming are utilized to explore pathway features that influence patient recovery time.	Identification of trajectories; Discovery of pathway features influencing the patients recovery time (prediction model)	Trajectory, Prediction	trajectory with weights as graph	ProM
Ku et al., 2020	Patient Pathway Analysis in Python; additional statistics; pathways of states;	Other		Discovery of temporal frequent patterns; Analysis of complexity, heterogenity of patient pathway	Proposal, Trajectory,	trajectory as graph - sankey, on timeline	Python, R
Lakshmanan et al., 2013	clustering to remove outliers (not obligatory); Pattern Mining using Sequential Pattern Mining with bitmap representation (SPAM); identification of patterns with high correlation with patients outcome using Information Gain	PM	relation between pattern and patient outcome (information Gain); users can overlay frequent patterns, ranked according to their correlation	Proposal of method to mine clinical care pathways correlated with health outcome	Proposal, Trajectory, Prediction	trajectory as graph; patterns in table with weights	BIP - business process insight, Java, google Web Toolkit

Additional File 2: Data Extraction of the included studies

Study	Applied method	Method - class	Prediction	Stated aim	Aim - class	Presentation of results	Program use
			with a particular patient outcome, on a mined model of the patient population with that outcome				
Lambert-Côté et al., 2020	Group based trajectory clustering; using statistical method	Clust.	Multinomial logistic regressions were used to identify factors associated with trajectories.	Identification of adherence trajectory groups; Explore factors associated with trajectories	Trajectory, Prediction	trajectory as graph, on timeline	STATA
Le Meur et al., 2015	Clustering. Similarity: Optimal Matching. Clustering: hierarchical with Wards linkage. State Sequence Analysis using TraMineR in R.<br>Additional statistics.	Clust.		Identifying disparitiers in care trajectories	Trajectory	trajectory as graph, on timeline	R
Le et al., 2019	Sequential Pattern Mining, including Variants, based on T-PrefixSpan; additional statistics	PM	no	Proposal of method to evaluate variants of Sequential Pattern; Consideration of safety and	Proposal, Pattern	pattern as graph	not named

Additional File 2: Data Extraction of the included studies

Study	Applied method	Method - class	Prediction	Stated aim	Aim - class	Presentation of results	Program use
				efficiency of sequences			
Li et al., 2019	reduce complexity; Sequential Pattern Mining with bitmap representation + temporal aspect	PM	Yes. Bag of Pattern construction for prediction	Proposal of method to mine patterns from sequence of point events; Evaluation of effectiveness and discriminative power	Proposal, Pattern	pattern in table with weights	Python
Meng et al., 2019	Construction of Line of Therapy; Clustering. Similarity: Euklidean distance of features. Clustering: partitioning, kmeans.	Clust.	Chi-square for relationships between clusters (membership) and treatment sequence	Proposal of method to derive patient level care trajectory; Derive temporal phenotypes of trajectories	Proposal, Trajectory, Pattern, Phenotyping	trajectory as graph - sankey	R
Najjar et al., 2018	grouping of complex objects to reduce complexity; Hierarchical clustering of hospital stays. Hidden Markov Model-Clustering, hierarchical and partitioning; Similarity: -; Clustering: k-prototype algorithm (hier.) for clustering of centers and hierarchical clustering with average linkage for final clustering.; Training of HMM in each cluster to obtain Model Parameters (Baum-Welch-Alg.)	Clust.		Proposal method to construct care pathway from administrative data; Construct clusters of pathways to analyze and describe them; Extraction of lattent patterns from patient care trajectories	Proposal, Trajectory, enable predictions	trajectory as graph - weighted	GHMM library, Scipy
Nuemi et al., 2013	construction of pathways; clustering of pathways: Partitioning methods (kmeans) and improbe	Clust.		Proposal of method to	Proposal, Trajectory, Phenotyping	trajectory in table with weights	SAS, ClustSYR software

Additional File 2: Data Extraction of the included studies

Study	Applied method	Method - class	Prediction	Stated aim	Aim - class	Presentation of results	Program use
	bayrcentres. Use of CluStsyr module of SYP software.			construct pathway profiles			(SYROKKO) + Adobe Illustrator CS 3; MapInfo
Oh et al., 2016	phenotyping patients on characteristics; conditional probabilities for comorbidities; further statistics attached	Other	To address the association between the different trajectories and the risk of developing diabetes, we constructed a multivariate logistic regression model for diabetes outcome using demographics […], glucose level, staged comorbidities […], and three trajectories.	Proposal of novel method to observe trajectories	Proposal, Trajectory, Prediction	trajectory in table with weights	R
Ou-Yang et al., 2019	grouping on fixed criterion; Generalized Sequential Pattern algorithm (GSP)	PM	association: disease pattern and medication prescribed with statistical analysis	Identification of sequential pattern of diseases, medication; diseases before Steven Johnson Syndrom	Pattern	pattern in table with weights	open-source data mining library

Additional File 2: Data Extraction of the included studies

**Study**	**Applied method**	**Method - class**	**Prediction**	**Stated aim**	**Aim - class**	**Presentation of results**	**Program use**
Perer et al., 2015	Sequential Pattern Mining with bitmap representation (SPAM); hierarchical clustering for vizualization; Use of Care Pathway Explorer	PM	relation between sequences and patient outcomes	Proposal of method by IBM; Identification of common sequences of care events	Proposal, Pattern	pattern as graph - sankey; pattern in table	python, DB2, Universal Feature Model (UFM) +D3.js
Pokharel et al., 2020	Sequential Pattern Mining with gap constrained in combination with Temporal Tree Technique. (Tree: on each level a sequence, following the branch events getting more detailled)	PM	Yes. KNN	Discovery of more complex frequent patterns	Pattern	trajectory in table; pattern in table with support	not named
Rama et al., 2019	Construction of discrete sequences. Hierarchical Clustering. Similarity: Needleman-Wunsch Algorithm. Clustering: Wards method; Bootstrapping for validation of clusters.	Clust.		Identify clusters of patients by analyzing longitudinal clinical data	Phenotyping	pattern as graph with weights	AliClu
Rao A. et al., 2018	grouping of events; Application of Sequence Analysis in R (TraMineR)	Other		Extraction of common sequences after surgery; Discovery of causes for readmission; Reduction of long-term readmission rate; readmission	Pattern, Prediction	pattern in table with weights	R
Rao A. et al., 2017	grouping of events; Application of Sequence Analysis in R (TraMineR)	Other		Identification of distinct sequences of emergeny readmission;	Pattern	pattern in table with weights	SAS, R

Additional File 2: Data Extraction of the included studies

Study	Applied method	Method - class	Prediction	Stated aim	Aim - class	Presentation of results	Program use
				emergency readmission			
Rao G. et al., 2018	transform data in pathform data; transition probabilies to extract common patterns - conditional probability	Other		Extraction of diagnostic paths	Trajectory	trajectory as graph - weighted	SAS
Righolt et al., 2020	Clustering of Patients. Similarity: (not specified); Clustering: kmeans (partitioning).	Clust.	no	Propsal of method to characterize longitudinal data	Proposal, (Trajectory), Phenotyping	trajectory as graph, on timeline	not named
Roux et al., 2018	State Sequence Analysis in R using TraMineR. Similarity: Optimal Matching. Clustering: Wards method (hierarchical).	Clust.		Proposal of a reference process regarding toe use of state sequence analysis; Analysis of care trajectories	Proposal, Trajectory	trajectory as graph, on timeline	R
Solomon et al., 2020	Markov Models for transition; prediction: log. Regression	MM	A supervised machine learning algorithm was then employed to determine longitudinal patient factors associated with TCZm use. Logistic regression model to	Identification of patient drugs pathways; Association of patient factors with medication use (TCZm)	Trajectory, Prediction	trajectory as graph, on timeline	SAS

Additional File 2: Data Extraction of the included studies

Study	Applied method	Method - class	Prediction	Stated aim	Aim - class	Presentation of results	Program use
			improve prediction (discriminate between patients on same treatment who move to different next stages/drugs)				
Sun et al., 2013	grouping of patients on characteristics; Sequential Pattern Mining	PM		Proposal of method to mine temporal dependency in outpatient encounter	Proposal, Pattern	pattern in table	not named
Vanasse et al., 2020	Multidimensional State Sequence analysis in R using TraMineR. Similarity: Optimal Matching. Clustering: Wards linkage criterion (hierarchical).	Clust.		Exploration of patterns care trajectories	Trajectory	trajectory as graph on timeline	not named
Vogt et al., 2017	Categorization of events, types of service and procedure; Clustering of patient sequences: Similarity: LCS, Clustering: Kmedoids (partioning). State Sequence Analysis in R using TraMineR. Additional statistics.	Clust.	We used logistic regression to identify the most effective sequences for avoiding hospitalizations.	Exploration of method to identify typical care sequences; Comparison of effectiveness of care sequences	Trajectory, Prediction	trajectory as graph on timeline	R, STATA
Wang et al., 2013	classification with nearest neighbors; temporal event signature mining using Stochastic Learning Scheme. Efficient onlie optimization schneme to make	Other	no	Proposal of novel representation, learning framework to find temporal signatures in longitudinal events; Extraction of event structure and relationship		pattern as graph	not named

Additional File 2: Data Extraction of the included studies

Study	Applied method	Method - class	Prediction	Stated aim	Aim - class	Presentation of results	Program use
	problem more scaleable; cNMF, sparsity regularization, beta-divergence.			with event sequence signature mining			
Wright et al., 2005	Sequential Pattern Mining (cSPADE)	PM	rules, support; 10-fold cross validation which medication was likely to prescribed next. PM for next medication prediction	Identification of relationships between medications and rules; Generation of rules to predict the medication prescribed next	Pattern, Prediction	pattern in table with weights	SQL, R + GraphViz
Yan et al., 2016	clustering of patients - similarity of block elements in refined sequences, k-means clustering; Software ProM algorithm to infer and vizualize workflow	Other	relation between subgroups and a range of diagnoses and procedures	Proposal of method to identify subgroups of Heart Failure through similarity analysis of event sequence	Proposal, Trajectory	trajectory as graph	not named + ProM (vizualization)
Zhang and Padman, 2015	clustering of patients (get patients cohorts) - hierachical clustering with LCS similarity, Silhouette for number of clusters; Markov Chain für Pathway;	MM	Yes. Viterbi in LOO-CV.	Proposal of a method to develop care trajectory from EHR	Proposal, Trajectory	trajectory in table	not named
Zhang, Padman, Wasserman, Patel, Teredesai and Xie, 2015	clustering of patient sequences (hierarchical: Wards method, similarity: LCS), cluster size as stopping criterion; Markov Chains for pathways per cluster	MM	Yes / No	Proposal of a novel method to automate clinical pathway learning process	Proposal, Trajectory	trajectory as graph - weighted	not named

Additional File 2: Data Extraction of the included studies

Study	Applied method	Method - class	Prediction	Stated aim	Aim - class	Presentation of results	Program use
Zhang, Padman and Patel, 2015	hierarchical clustering (dLCS similarity, hierarchical) and definition of super nodes to reduce complexity; Makov Model for sequences.	MM	No	Proposal of a method for pathway development; Extraction of common patient centered pathways from EHR	Proposal, Trajectory	trajectory as graph - weighted	not named
Zhang et al., 2014	Presentation of visit sequences. Clustering of sequences. Similarity: LCS distance. Clustering: hierachical (not specified); Optimal number of clusters: Silhouette.	Clust.		Analysis of treatment data; Learning of practice-based clinical pathways	Trajectory	trajectory as graph - weighted	not named + gephi (vizualization)
